# Patient flow in emergency departments: a comprehensive umbrella review of solutions and challenges across the health system

**DOI:** 10.1186/s12913-024-10725-6

**Published:** 2024-03-05

**Authors:** Mahnaz Samadbeik, Andrew Staib, Justin Boyle, Sankalp Khanna, Emma Bosley, Daniel Bodnar, James Lind, Jodie A. Austin, Sarah Tanner, Yasaman Meshkat, Barbora de Courten, Clair Sullivan

**Affiliations:** 1 Faculty of Medicine, Centre for Health Services Research, The University of Queensland, Brisbane, Australia; 2 Faculty of Medicine, Queensland Digital Health Centre, The University of Queensland, Brisbane, QLD 4072 Australia; 3 Princess Alexandra Hospital, Brisbane, Australia; 4 Faculty of Medicine, The University of Queensland, Brisbane, Australia; 5 The Australian E-Health Research Centre, Commonwealth Scientific and Industrial Research Organisation, Brisbane, Australia; 6 Queensland Ambulance Service, Queensland Government, Brisbane, Australia; 7 Gold Coast University Hospital, Gold Coast, Australia; 8 Department of Health, Metro North Hospital and Health Service, Brisbane, Australia; 9 School of Health and Biomedical Sciences, RMIT University, Melbourne, Australia

**Keywords:** Patient flow, Emergency department, Solutions, Intervention, Outcomes, Challenges, Umbrella review

## Abstract

**Background:**

Globally, emergency departments (EDs) are overcrowded and unable to meet an ever-increasing demand for care. The aim of this study is to comprehensively review and synthesise literature on potential solutions and challenges throughout the entire health system, focusing on ED patient flow.

**Methods:**

An umbrella review was conducted to comprehensively summarise and synthesise the available evidence from multiple research syntheses. A comprehensive search strategy was employed in four databases alongside government or organisational websites in March 2023. Gray literature and reports were also searched. Quality was assessed using the JBI critical appraisal checklist for systematic reviews and research syntheses. We summarised and classified findings using qualitative synthesis, the Population-Capacity-Process (PCP) model, and the input/throughput/output (I/T/O) model of ED patient flow and synthesised intervention outcomes based on the Quadruple Aim framework.

**Results:**

The search strategy yielded 1263 articles, of which 39 were included in the umbrella review. Patient flow interventions were categorised into human factors, management-organisation interventions, and infrastructure and mapped to the relevant component of the patient journey from pre-ED to post-ED interventions. Most interventions had mixed or quadruple nonsignificant outcomes. The majority of interventions for enhancing ED patient flow were primarily related to the 'within-ED' phase of the patient journey. Fewer interventions were identified for the 'post-ED' phase (acute inpatient transfer, subacute inpatient transfer, hospital at home, discharge home, or residential care) and the 'pre-ED' phase. The intervention outcomes were aligned with the aim (QAIM), which aims to improve patient care experience, enhance population health, optimise efficiency, and enhance staff satisfaction.

**Conclusions:**

This study found that there was a wide range of interventions used to address patient flow, but the effectiveness of these interventions varied, and most interventions were focused on the ED. Interventions for the remainder of the patient journey were largely neglected. The metrics reported were mainly focused on efficiency measures rather than addressing all quadrants of the quadruple aim. Further research is needed to investigate and enhance the effectiveness of interventions outside the ED in improving ED patient flow. It is essential to develop interventions that relate to all three phases of patient flow: pre-ED, within-ED, and post-ED.

**Supplementary Information:**

The online version contains supplementary material available at 10.1186/s12913-024-10725-6.

## Background

Changes in demographics, the prevalence of multimorbidity, the ongoing challenges posed by the COVID-19 pandemic, and persistent shortages in healthcare staffing have significantly increased the demand for healthcare services. [1–3]. Most hospitals face a mismatch between supply and demand, resulting in delays, staffing gaps, and inefficient hospital ward utilisation. This imbalance leads to issues such as overcrowded emergency units, nursing staff shortages, and staff dissatisfaction. [3]. Globally, emergency departments are overcrowded and unable to meet an ever-increasing demand for healthcare [1]. The increasing demand for emergency care services is a significant challenge for healthcare systems worldwide [4–9].

Patient flow through the healthcare system refers to the movement of patients through care settings and encompasses the entire patient journey from arrival until the patient departs from the hospital [7, 10, 11]. Effective patient flow is essential for timely, high-quality care, and mismanagement can cause disruptions within the hospital system [3, 9, 12]. Poor patient flow can lead to ED overcrowding when patients experience delays or blockages in the care processes [9].

Access block, defined as the delay of admitted patients from leaving the ED for more than eight hours due to a shortage of hospital beds, is a significant cause of poorer patient outcomes [13]. It specifically impacts admitted patients and is different from ED overcrowding, which affects both admitted and nonadmitted ED patients [13–15]. ED overcrowding and access block have numerous negative consequences, such as decreased quality of care, poor patient outcomes, increased risk of death, medical errors, treatment side effects, patient dissatisfaction, reduced hospital capacity, ambulance diversions, increased workload, staff frustration, longer waiting times, increased cost of care, and patients leaving without being seen by a physician. The negative consequences of access block and overcrowding highlight the urgent necessity for interventions to tackle these issues and improve patient outcomes while maximising healthcare system capacity [1, 9, 16–19].

Studies have shown that addressing patient flow issues can have several benefits, including reduced length of stay (LOS) and faster discharge process [1, 2, 20, 21]. Improving patient flow is essential for enhancing medical quality, safety, and patient satisfaction while also reducing input and facilitating throughput and output from hospitals and EDs [3, 8, 22].

It has become evident that ED overcrowding is not solely an ED problem but rather reflects dysfunction throughout the entire patient journey. However, reform efforts and political pressure traditionally focus on ED processes [23, 24]. Healthcare aims have evolved over time, with measures of ED performance traditionally centred around process measures such as ED length of stay, to now reach for the more balanced Quadruple Aim of Healthcare: increased efficiency, improved population health, better consumer experience and excellent provider experience [25]. Balanced measures of healthcare performance, including experience and care outcomes, are important. Focusing solely on LOS without measuring experience or outcomes can lead to clinician burnout and low-value or dangerous care [26].

Despite extensive literature addressing patient flow interventions and challenges, stakeholders may find it challenging to navigate and determine evidence-based interventions and challenges strongly supported by the evidence. [7, 27–29], Syntheses of research on how to improve patient flow rarely provide an overall examination of interventions across the care pathway [13, 30–36]. Decision-making in a holistic manner to improve patient flow can be a difficult, complex, and potentially risky task for stakeholders [27, 28].

Existing reviews on this topic typically focus solely on interventions based in the ED to improve patient flow within the ED [1, 7, 27, 28, 37–40]. However, there is currently a noticeable gap in recent reviews that comprehensively address challenges and interventions across the health system for managing ED patient flow. Recognising the importance of a broader health-wide perspective, extending from pre-ED to discharge and beyond, is crucial. This emerging concept requires a holistic approach that views the healthcare system as a continuum of care [41]. Therefore, this review aims to synthesise the literature comprehensively, focusing on evidence-based interventions throughout the entire hospital or health system to enhance ED patient flow. Additionally, it explores outcomes related to ED patient flow improvement and identify challenges within the entire hospital or health system interconnected with the patient flow in the ED, recognising the healthcare system as a continuum of care.

In our study, we utilised the population–capacity–process (PCP) model of health service design. According to this model, effective services establish a connection between a defined population, the necessary capacity, and a streamlined process [42, 43]. The term 'Population' refers to individuals with shared needs, 'capacity' pertains to the human and physical resources needed to meet those requirements, and 'process' encompasses the steps that bridge the gap between the two. This model emerged from a study highlighting that the failure of patient-flow initiatives often results from neglecting one or more of these three domains. Subsequently, the PCP model has found application in other literature [8, 42–45].

In addition, we applied the Input/Throughput/Output (I/T/O) model of ED patient flow, developed by Asplin et al., which divides ED crowding into three interconnected components: input, throughput, and output [10]. This conceptual framework aids administrators, researchers, and policymakers in comprehending the causes of ED crowding and developing potential solutions.

## Methods

An umbrella review was conducted to comprehensively summarise and synthesise the available evidence from multiple research syntheses on various challenges and interventions within the entire health system, specifically focusing on patient flow in the ED. This umbrella review followed the Joanna Briggs Institute methodology for umbrella reviews [46, 47] and the PRIOR checklist for healthcare overviews to ensure thoroughness and transparency [48]. This umbrella review followed an a priori published protocol and was registered with the International Prospective Register of Systematic Reviews (PROSPERO) on 24 April 2023 (CRD42023414182) [49].

### Inclusion and exclusion criteria

As this study reviewed both quantitative and qualitative systematic reviews, we considered both interventions and phenomena of interest when defining eligibility criteria for this umbrella review. We used PICO (population, intervention, comparator, outcome) or PICo (population, phenomena of interest and context) elements to clearly define the eligibility criteria.

#### Types of participants

Participants included consumers of health care services, physicians, nurses, health care professionals, health care workers in clinics, hospitals, ambulance service, primary care, and residential aged care facilities (RACF), carers, health managers and policymakers. Participants who were not responsible for patient flow-related activities, such as healthcare workers without a direct role in patient care or management, were excluded.

#### Interventions/Phenomena of interest

The interventions reviewed in this study included interventions or potential solutions throughout the entire health system aimed at improving patient flow in the ED. The phenomena of interest were the challenges and root causes, encompassing both internal and external organisational factors, that hinder efficient patient flow in the ED. Interventions or phenomena of interest that were not related directly or indirectly to patient flow in the ED were excluded.

#### Comparator(s)/Context

This umbrella review aimed to synthesise evidence on challenges and potential solutions throughout the entire health system with a focus on ED patient flow without making direct comparisons between interventions. However, the review considered studies that compared patient flow interventions or strategies against each other or against usual care or no intervention. Studies comparing interventions or exposures unrelated to patient flow in the ED were excluded.

Interventions were not limited to the ED, and any intervention that measured an impact on ED flow was considered.

#### Outcomes

To provide a balanced overview of the evidence base related to the topic, this review attempted to report both beneficial and adverse outcomes of interventions across the entire health system aimed at improving patient flow in the ED and map them to the Quadruple Aim. Studies that reported outcomes unrelated to interventions or challenges of patient flow in the ED were excluded.

#### Types of studies

The systematic reviews included in our study were needed to use internationally accepted methodologies such as meta-analyses, qualitative systematic reviews, integrative reviews, scoping reviews, meta-syntheses, and meta-aggregative reviews. In addition, umbrella reviews that reviewed quantitative, qualitative, or both quantitative and qualitative systematic reviews were included. We excluded primary studies, as well as narrative reviews, systematic reviews based on theoretical studies or opinions, editorials, commentaries, predictive studies, and feasibility studies.

### Search strategy

Two authors (MS and CS) developed the search strategy, which was then peer-reviewed by an experienced librarian (LE) in accordance with the Peer Review of Electronic Search Strategies (PRESS) 2015 Guideline Statement for systematic reviews [50].

To develop a comprehensive search strategy, several steps were taken [51]. The first step involved conducting a preliminary search in PubMed to identify additional keywords and synonyms relevant to the initial keywords. The initial keywords used were “patient flow”, "emergency department," "emergency care," and "systematic review." Subsequently, a search strategy was developed that included appropriate search terms and Boolean operators (such as "AND" and "OR"), along with MeSH and Emtree terms. After piloting the search strategy in PubMed and making necessary adjustments, the final version of the search strategy was developed. Table 1 presents our final search strategy. The search strategy for each database was subsequently developed according to the specific syntax and indexing of that database (Additional file 1).

**Table 1:** Search strategy

NO	Construct	
#1	"Emergency Service, Hospital" OR "Emergency Medicine" OR "Emergency Nursing" OR "emergency medicine" OR "emergency nursing" OR "Hospital Emergency Service" OR "Hospital Emergency Services" OR "Emergency Hospital Service" OR "Emergency Hospital Services" OR "Emergency Department" OR "Emergency Departments" OR "Emergency Unit" OR "Emergency Units" OR "Emergency Ward" OR "Emergency Wards" OR "Emergency Room" OR "Emergency Rooms" OR "trauma center" OR "trauma centers" OR "trauma unit" OR "trauma units" OR (emergency AND hospital)	
#2	"crowding" OR crowd* OR congest* OR overcrowd* OR gridlock* OR queu* OR overload* OR "access block*" OR "Patient flow" OR "patient inflow" OR "Patient turnover" OR "patient Caseload" OR "patient Caseloads" OR "patient throughput*" OR "emergency department throughput" OR "patient journey" OR "patient inflow" OR "patient path*" OR "patient disposition" OR "patient dispositions" OR bottleneck OR bottlenecks OR challenge OR challenges OR barriers OR barrier OR "Patient boarding" OR delay OR delays OR"choke point" OR "choke points"	
#3	“systematic review” OR “systematic reviews” OR meta anal* OR meta-anal* OR meta syn* OR meta-synth* OR systematic OR scoping	
#4	#1 AND #2 AND #3	Limits: English language 2018/1/1—2023/3/3

In March 2023, we conducted an extensive search in electronic databases, which included CINAHL, PubMed, Web of Science, and Embase. Additionally, we simply conducted a basic search of major systematic review repositories, such as the JBI Database of Systematic Reviews and Implementation Reports, the Cochrane Database of Systematic Reviews, and the PROSPERO register.

Reports on government or organisational websites are eligible for inclusion in an umbrella review and can help decision-makers base their decisions on evidence [52]. As such, we searched for grey reports on relevant government or organisational websites, Google, and Google Scholar. Finally, the reference lists of all included systematic reviews were searched for additional relevant publications.

### Study screening and selection

The retrieved references were imported into EndNote v.20.4.1 (Clarivate Analytics, PA, USA), and duplicates were removed. The remaining citations were subsequently uploaded to Covidence Systematic Review Software (Veritas Health Innovation, Melbourne, Australia) for screening, methodological quality appraisal and data extraction.

To enhance consensus among reviewers, a pilot selection process was conducted on a randomly selected 3% of articles. Subsequent minor revisions were made to the eligibility criteria. Two independent reviewers screened the titles and abstracts of all potentially eligible studies, followed by a review of the full text of those that met the initial screening criteria. In cases where disagreements arose, a third reviewer was brought in to resolve disputes that could not be resolved through consensus. We used the PRISMA flowchart to describe the process of study selection in the Results section. The comprehensive details regarding systematic reviews that were excluded after this assessment are presented in Additional file 2.

### Assessment of methodological quality

In the present study, two independent reviewers assessed the reviews included within the analysis. The JBI critical appraisal checklist for systematic reviews and research synthesis was utilised to assess the trustworthiness, quality, and research findings of the articles, which was prepared in Covidence. This checklist comprises eleven distinct aspects, and the appraisal of these aspects was conducted using four criteria: "yes", "no", "unclear", and "not applicable" (Additional file 3). Using the JBI critical appraisal toolkit, each of the included studies was categorised into one of three quality levels: low, moderate, or high [52]. The classification criteria categorised a paper as "low quality" if its results were below 50%, "moderate quality" if they ranged between 50 and 69%, and "high quality" if the results were above 69%. Any disagreements between the reviewers were resolved through discussion and consensus within the research team. Our decision was to include all reviews in our study without any prior exclusion based on quality assessment and to provide the results of all quality appraisals. This differs from the JBI methodology, which suggests setting a quality score cut-off [47]. However, as our umbrella review aimed to examine the quality of systematic reviews, we believe it is crucial to present data on all the reviews we have included and enable readers to assess the value of information provided by each systematic review.

### Data collection

In this study, two reviewers independently extracted data from the included reviews using the modified JBI data extraction form for systematic reviews and research syntheses [47]. The data extraction form was customised for the purpose of this research and developed in Covidence (Additional file 4). To enhance the clarity, relevance, accuracy, and consistency of data extraction, three reviewers independently piloted the form on a randomly selected 10% of the included studies, which consisted of two narrative syntheses and two meta-analyses. Any potential revisions to the data extraction tool were assessed by all reviewers and discussed in detail before extracting data independently.

Data extracted included citation details, objectives, type of study, description of participants, setting and context, search details, appraisal data, key findings related to integration interventions and challenges, and any comments or notes from the umbrella review authors regarding any included study.

### Data synthesis

Four authors (CS, MS, ST, YM) analysed the data extracted to develop a narrative overview of the challenges and interventions in patient flow. Given the heterogeneity in populations, outcomes, and analyses, we summarised the findings of the included reviews using a narrative synthesis approach. Qualitative research synthesis was used, following the guidelines for conducting an umbrella review [46].

A meta-aggregative approach was employed to synthesise qualitative evidence of challenges regarding challenges in patient flow across the healthcare system. The goal was to generate evidence that can guide practitioners and policymakers [47, 53]. The findings on challenges were categorised based on the PCP model.

We also utilised an inductive approach to synthesise evidence on patient flow improvement solutions and gain a comprehensive understanding of applied interventions. Additionally, to provide a clear and structured framework, we adopted the input/throughput/output (I/T/O) model of ED patient flow. [54]. We also used the 'pre-ED', 'within-ED', and 'post-ED' model, as employed by the Sax Institute to describe solutions for reducing access blocks across the health system [13]. 'Input' refers to interventions or challenges before patients arrive in the ED, 'throughput' refers to internal ED issues and interventions while the patient is in the ED, and 'output' refers to interventions and challenges of the patient's journey in leaving and after leaving the ED. Post-ED was classified into three potential pathways: home departure, ward departure, or transfer to a residential care facility. The outcomes of the interventions were indicated using specific symbols: ( +) for positive outcomes, (-) for negative outcomes, ( ±) for mixed outcomes or conflicting evidence, ( =) for nonsignificant outcomes or no difference, and (NR) for not reported or limited evidence.

Additionally, the extracted outcomes of interventions for each component were synthesised and classified based on the Quadruple Aim framework. These outcomes were aligned with the Quadruple Aim framework, which includes QAIM1: improving the patient experience of care, QAIM2: improving population health, QAIM3: reducing costs, and QAIM4: enhancing the work-life balance and satisfaction of healthcare providers.

## Results

### Study selection

**Fig. 1:**
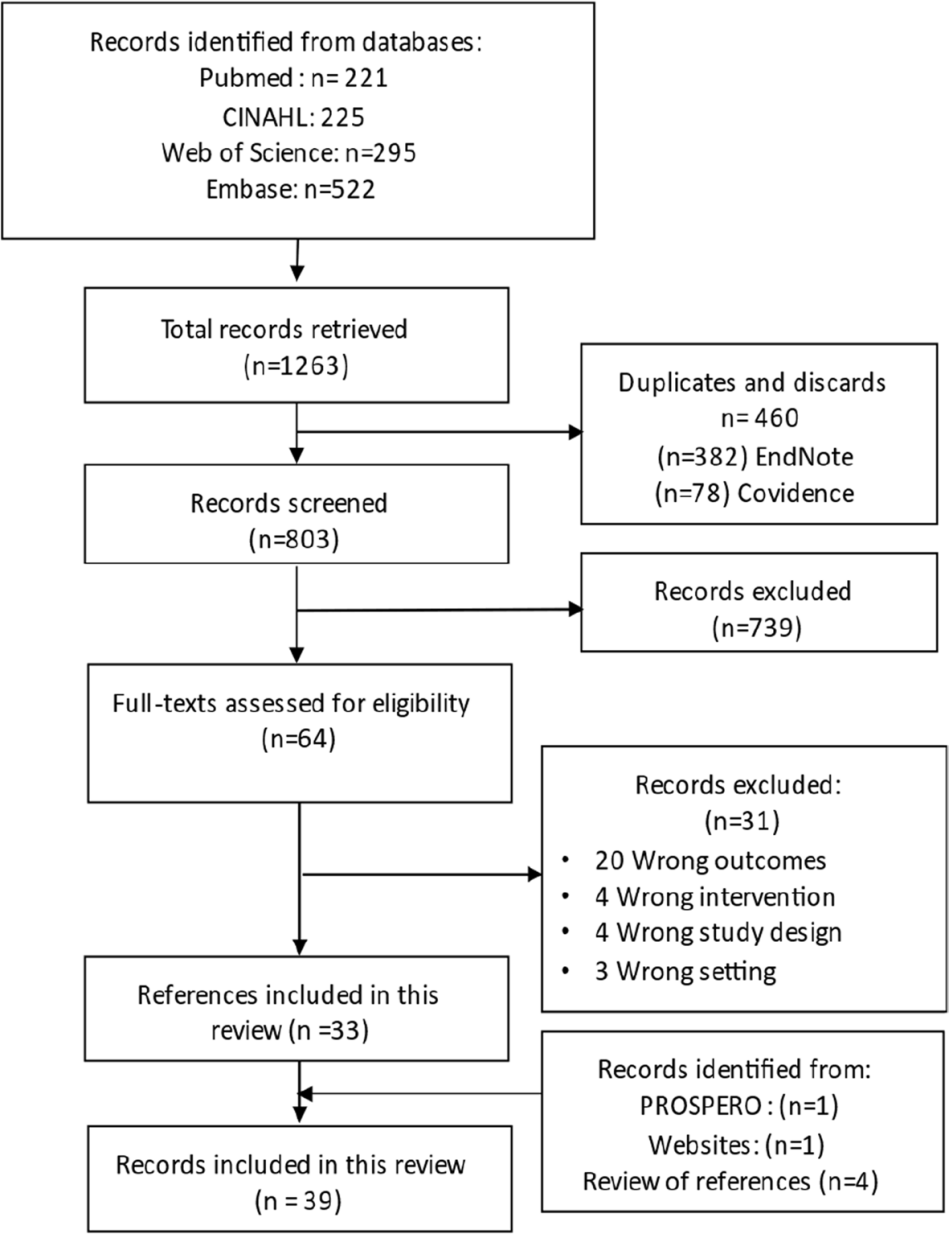
Flowchart detailing the identification and selection of research syntheses for inclusion in the umbrella review

Figure 1 provides an overview of the flow diagram representing the study selection process. Through the literature search, a total of 1263 titles were retrieved. The search in gray literature, PROSPERO databases, and the reference lists of eligible articles yielded 6 additional records. After removing duplicates (*n* = 460) and screening titles and abstracts, 64 records were considered eligible for full-text review. A total of 39 articles were selected against the selection criteria and included in the literature review.

### Description of the included reviews

The general characteristics of the included reviews are presented in Table 2. These reviews cover various topics related to ED interventions, access blocks, patient flow, and healthcare outcomes. The total number of included reviews was 39, and they were conducted between 2017 and 2023, with a significant proportion (*n* = 12) published in 2020. The studies included in these reviews span a wide range of years, from 1980 to 2022, indicating a comprehensive examination of the literature over an extended period. The included studies comprised 8 scoping reviews, 24 systematic reviews, 3 systematic reviews with meta-analyses, 3 umbrella reviews, and 1 systematic mapping review, representing a diverse range of study designs. This varied selection of methodologies provided a comprehensive and well-rounded examination of the research topic. Among the included reviews, 7 conducted quantitative analysis or meta-analysis, 4 performed narrative synthesis and meta-analysis, and 28 reviews presented the results of the primary studies narratively or descriptively. Full details of the characteristics of the included reviews are available in Additional file 5. The various appraisal instruments and ratings used in the included reviews. The appraisal instruments mentioned include the ROBIS tool, SQUIRE 2.0 checklist, EPHPP, GRADE, Newcastle‒Ottawa Scale Modified tool, AMSTAR 2, modified 7-level rating system, JBI checklists, Cochrane EPOC, NHLBI, NICE quality appraisal tool, EBL critical appraisal tool, RevMan ROBIS tool, QATSDD, SIGN, and CAS Pand MINORS.

**Table 2:** Summary characteristics of included reviews

Author, Year country (Ref No)	Aim/Objective	Study design	Participants (Number)	Number (range of studies included)	Types of studies included	Country of origin of incl. studies	Sources searched	Appraisal instruments / rating	Method of synthesis
AM, 2022 Australia [13]	Solutions to access block; present recommendations for evidence-based access block solutions for piloting and/or implementation in Australia and Aotearoa New Zealand	ScR	Not reported—however patients in ED	Retrieved: 199 Included: not clear (2000–2022)	Qualitative Quantitative studiSes	Australia, Europe, North America, Japan, Taiwan, Hong Kong, Singapore, South Korea, South America	CINAHL, Medline, Embase, WOS	Not appraised	Not stated- however narrative synthesis
Austin, 2020 Australia [30]	To map the research evidence provided by reviews on strategies to measure and improve ED performance	ScR	Clinicians, patients, and/or administrators in the ED	77 (2000–2019)	SR PR UR IR CR Subs R RR SCR EBR, NR, Mas	Denmark, Canada, USA, England, Australia, Hong Kong, Sweden, Italy, Iran, New Zealand, Brazil, South Korea, Switzerland	Cochrane, Scopus, Embase CINAHL, PubMed	-ROBIS tool 1 low bias, 31 high bias, 15 unclear bias	Narrative synthesis
Beckerleg, 2020 Canada [31]	To identify what interventions have been tried to reduce consultation to decision time and, in turn, ED length of stay	SR	Adult patients (> / = 18 years of age)	9 (2011–2018)	RCR RBAs PBAs	South Korea, Canada, USA	MEDLINE, EMBASE, Cochrane, CINAHL	SQUIRE 2.0 checklist 3 poor, 5 fair, 1 good	Descriptive qualitative analysis
Benabbas, 2020 USA [55]	To ascertain the role of triage liaison providers in improving throughput metrics to minimise patient risk and improve safety and quality metrics set forth by CMS	SR & Mas	Patients in the ED (329,340)	12 (2001–2019)	RBAs PBAs CBAs	USA	PubMed, EMBASE, WOS, www.ntis.gov, clinicaltrials.gov, SAEM, ACEP, AAEM, Opengrey, Google scholar	EPHPP 66.7% moderate, 33.3% weak	Meta-analysis
Berning, 2020 USA [32]	To summarise interventions that impact the experience of older adults in the ED as measured by patient experience instruments	SR	Older adults (3163)	21 (1996–2018)	RCT QE Obs B & A	USA UK Australia Sweden, Canada Korea Scotland	Ovid Central, Ovid EMBASE, Ovid, MEDLINE PsycINFO, Clinicaltrials	GRADE Newcastle‒Ottawa Scale Modified tool GRADE: ROBIS tool: 13 high, 4 moderate, 4 low	Qualitative analysis
Bittencourt, 2020 Brasil [27]	To present an overview of systematic reviews on throughput interventions to solve the overcrowding of emergency departments	UR	N/A	15 (2007–1016)	SR	Australia, Netherlands, USA, Italy	PubMed, Cochrane, EMBASE, Health Systems Evidence, CINAHL, SciELO, LILACS, CAPES portal, Google scholar	AMSTAR 2 3 critically low, 4 low, 6 moderate, 2 high	Narrative synthesis
Blodgett 2021 UK [56]	To identify all studies that examined alternate routes of care for the nonurgent "intermediate" patient instead of ED conveyance	ScR	N/A	41 (2000–2020)	Qualitative Quantitative Consensus-base (Commentaries Protocols Policy)	UK, Sweden, Netherlands, USA, Australia, Canada, Ireland	PubMed, CINAHL, WOS ProQuest NHS Evidence, CORE, BL.UK, Open-Grey, HMIC	Modified 7-level rating system for the hierarchy of evidence 2 Level 2, 4 Level 3, 11 Level 4, 13 Level 6, 11 level 7	Narrative synthesis
Boylen 2020 Australia [57]	To identify, critically appraise and synthesise evidence on the impact of professional interpreters on outcomes for hospitalised children from migrant and refugee families with limited English proficiency	SR	Limited-English-proficient migrant, refugee or asylum-seeker families with a hospitalised child used a professional interpreter (1813 families)	6 (2004–2018)	Quantitative RCTs QE and descriptive	USA	CINAHL Plus, Ovid,PubMed, ProQuest, Scopus, WOS, Embase, PsycINFO, Science Direct, APAIS Health, AIHW, AustHealth, Factiva, TROVE & OIASTER, Google Scholar	JBI checklists, JBI SUMARI, GRADE 4 Moderate, 2 high	Narrative synthesis
Brambilla 2022 Italy [58]	To shed light on the Free Standing Emergency Department (FSED) model and compare it with the traditional Hospital Based Emergency Department (HBED) in international contexts	SR	N/A	23 (2010–2021)	LR, CS, Theoretical studies, Applied studies	USA, France, Spain, Sweden, Italy	CINAHL, Scopus, PubMed, WOS	Not appraised	Narrative synthesis
Burgess 2021 Australia [33]	What is the effectiveness of nurse-initiated interventions on patient outcomes in the emergency department?	SR	All patients accessing treatment in an ED setting; (12 studies paediatric patients only, 14 studies adult patients only) (9144)	26 (2000–2019)	RCT, QE	Australia, USA, Netherlands, Hong Kong, Sweden, Canada, Iran, Saudi Arabia	PubMed, CINAHL, Embase, PsycINFO, WOS, Cochrane Central, Register of Controlled Trials, ProQuest Mednar	JBI checklists for experimental and quasiexperimental studies 4 < 50%, 1 50—69%, 17 > 69%	Quantitative Analysis (Meta-analysis) & Qualitative analysis
Cassarino 2019 Ireland [59]	To synthesise the totality of evidence relating to the impact of early assessment and intervention by Health and Social Care Professional teams on quality, safety, and effectiveness of care in the ED	SR	adults > / = 65 years old who present to the ED (273,886)	6 (2002–2013)	nRCTs CBAs ITS RMS	Australia	CINAHL, Embase, Cochrane Library, MEDLINE	Cochrane EPOC 6 high risk of selection bias and 6 unclear/high risk of performance bias	Narrative/ qualitative synthesis
Clark 2022 Australia [34]	To lead one through the patient journey and explore scholarly solutions from a diverse body of literature and methodologies to address bottlenecks in access and care delivery	ScR	N/A	43 (215–2021)	Quantitative, Qualitative mixed-methods, NR, ScR, Discussion papers	Trinidad, Kuwait, Iran, Canada, USA, Australia, New Zealand, Europe, France, China, Sweden, UK	CINAHL, Embase, ProQuest, PubMed, Cochrane Library	Not appraised	Narrative synthesis
DeFreitas 2018 UK [7]	To provide a comprehensive analysis of the evidence from existing systematic reviews on the interventions that improve ED patient flow	UR	N/A	13 (2006–2016)	SR	USA, Sweden, Australia, Canada, Korea, France, Germany, New Zealand, Saudi Arabia, Singapore, Switzerland, Turkey, Jamaica	Ovid, EMBASE CINAHL, Cochrane library, JBI, ProQuest, Open Grey, Google Scholar	AMSTAR 2 5 high, 3 Moderate, 3 Low, 2 Critically, low	Narrative synthesis
DiLaura 2021 Italy [35]	To review the literature on the issues encountered in the efficiency of EDs worldwide	SR	N/A	28 (2010–2019)	Obs, Modelling studies, QE, CRT	USA, Australia, Canada, China, France, Italy, Portugal, Sweden, Netherlands, Germany, UK	PubMed, Scopus, Cochrane library	Not appraised	Descriptive analysis
Franklin 2022 USA [60]	To characterise the evidence related to hospital capacity command centers and synthesises current data regarding their implementation	ScR	N/A	8 (2015–2019)	nCBAs	USA	PubMed, ABI ProQuest, Grey literature sources	Not appraised	Descriptive synthesis
Gonçalves-Bradley 2018 UK [61]	The aim of this Cochrane Review was to determine whether placing primary care professionals, such as general practitioners, in the hospital ED to provide care for patients with nonurgent health problems can decrease resource use and costs	SR	11,463 patients, 16 GPs, 9 eNPs, 69 emergency physicians	4 (1995–2015)	RCT nRCT B & A TS	Australia, Ireland, UK	Cochrane library, MEDLINE, Embase, CINAHL, PsycINFO, Grey literature, WHO ICTRP, Clinicaltrials.gov	GRADE EPOC 4 very low EPOC: high risk of bias	Quantitative Analysis (Forest plots without summary estimates)
Gottlieb 2021 USA [62]	To review the medical literature to determine the utility of triage-ordered testing and to offer evidence-based recommendations to emergency physicians	SR	Triage nurse, ED patients (Not reported)	13 (1990–2018)	RCTs, SR, Obs	USA UK Australia Sweden, Canada, China, HongKong	PubMed	A grade of evidence 1 outstanding, 4 good, 5 adequate, 3 poor	Narrative synthesis
Gottlieb 2021 USA [63]	Effect of Medical Scribes on Throughput, Revenue, and Patient and Provider Satisfaction: A Systematic Review and Meta-analysis	SR & MA	Medical scribes, ED patients 562,682 patient encounters	39 (2010–2020)	RCT, nRCT, RBAs, PBAs	USA, Canada, Australia	PubMed, Scopus, the Cumulative Index of Nursing and Allied Health Literature, Latin American and Caribbean Health Sciences Literature database, Google Scholar, Cochrane library, ACEP, SAEM CAEP	Newcastle‒Ottawa scale, Cochrane ROBIS Tool, GRADE criteria Studies were deemed to be good quality overall	Meta-analysis
Grant 2020 Canada [64]	To evaluate and summarise the results of studies describing ED throughput interventions	SR	N/A	101 (1996–2020)	RCT, B & A Cohort, CS, Obs, RCR	USA, Canada, Sweden, Australia, Netherlands, Spain, UK, Turkey, Pakistan, Finland, Korea, Jamaica, Taiwan	Medline, Embase, CINAHL, Cochrane Central	Cochrane ROBIS tool, NHLBI 19 good, 67 fair, 5 poor (NHLBI) 3 low, 5 some concerns (Cochrane)	Narrative synthesis Meta-analysis
Grant 2020 Canada [65]	To review, categorise and evaluate interventions to reduce preventable long-term care facility transfers to ED	ScR	Residents in long term care facilities (80,791 sample size)	26 (1988–2018)	RCT, Obs, Cohort, QE, B&A, RCT, cross-ectional, TS study, nRCT	Not reported	Medline, EMBASE, CINAHL	NHLBI 11 good, 14 fair, 1 poor	Narrative synthesis
Hesselink 2019 Netherlands [66]	Effectiveness of interventions to alleviate ED crowding by older adults: a systematic review	SR	Older adults (≥ 60 years of age) (Not reported)	16 (1996–2006)	RCT, nRCT, CBAs	US, Canada, UK, Australia, Singapore	CINAHL, Cochrane Library, EMBASE, PsychInfo, Grey literature,	Cochrane EPOC 12 high risk, 2 low risk, 2 moderate risk of bias	Quantitative Analysis
Hong 2020 Canada [36]	To understand the association between improved access to after-hours primary care and both ED and primary care utilisation	SR	N/A	20 (2000–2020)	cross-sectional, B&A	US, Australia, Belgium, England, Ireland, the Netherlands, Canada, Italy, Scotland	CINAHL, EMBASE, MEDLINE, Scopus, Google Scholar	Not appraised	Descriptive Synthesis
Hughes 2019 USA [67]	To evaluate the effect of ED interventions on clinical, utilisation, and care experience outcomes for older adults	SR	Older adults > / = 65 Not reported	17 references (15 unique studies) (1996–2017)	RCT, nRCT, CBAs	OECD countries: Australia, Canada, Europe, USA	PubMed, Embase, CINAHL, PsycINFO, ClinicalTrials.gov, Scopus	Cochrane EPOC,RADE For Objective outcomes: 7 high risk, 3 unclear, 4 low risk, 1 NA	Quantitative synthesis when was possible,
Jeyaraman 2022 Canada [68]	Impact of employing primary healthcare professionals (PHCPs) in ED triage on patient flow outcomes: a systematic review and meta-analysis	SR & MA	PHCPs, ED patients	40 (1993–2020)	B&A, RCT, Cohort studies, CBAs, QE, Cross-sectional, Obs	Saudi Arabia, Oman, UK, USA, Canada, Australia, France, China, Netherlands	Ovid, Cochrane Library, CINAHL	NICE quality appraisal tool 33 low quality, 7 moderate quality	Meta-analysis
Jeyaraman 2021 Canada [69]	To conduct a scoping review to identify and summarise the literature on interventions involving primary healthcare professionals to manage ED overcrowding	ScR	N/A	268 studies (274 reports) (1981–2020)	RCTs, nRCT, Cohort studies, CS, Cross-sectional, B&A, TS, Mixed-methods	USA, UK, Canada, Australia, Netherlands, Switzerland, Sweden, France, Italy, South Korea, China, New Zealand, Saudi Arabia, Taiwan, Belgium, Brazil, Finland, Oman, Portugal, Spain, Singapore	Ovid, Cochrane Library, CINAHL, CFHI, IHI website, AHRQ website, NHS Improvement, ISQUA, Quality Ontario, Saskatchewan Health Quality, HQCA, BCPCQC, Australian Commission on Safety and Quality in Health Care, HQSC New Zealand	Not appraised	Descriptive statistical analyses
Kirkland Canada 2019 [70]	To examine the effectiveness and safety of prehospital and ED-based diversion strategies on ED utilisation, non-ED healthcare utilisation and patient outcomes compared with standard emergency care responses	SR	Low-acuity ED patients Not reported	15 (2002–2017)	RCT, Cohort studies	England, USA, Scotland, Wales, Sweden	Medline, Embase, Cochrane Library, PsycINFO, CINAHL, Social Services Abstracts, ProQuest, Google Scholar, ClinicalTrials.gov, SAEM, CAEP	Newcastle‒Ottawa Scale, Cochrane ROBIS tool 10 high or unclear, 5 moderate	Meta-analyses
Leduc 2021 Canada [71]	To identify existing programs where allied healthcare personnel are the primary providers of the intervention and to evaluate their effectiveness and safety	SR	Adult patients living in long term care centres Not reported	22 (2013–2018)	RCTs, Obs	United States, Canada, Scotland, Norway	Medline, Embase, CINAHL Grey literature: clinicaltrials.gov, PROSPERO, CENTRAL	Cochrane ROBIS tool, Ottawa scale 2 high risk, 1 low, 1 some concerns, observational studies range: 2 – 9	Narrative synthesis
Malik 2018 Ireland [57]	To systematically review the impact of geriatric focused nurse assessment and intervention in the ED on hospital utilisation in terms of admission rate, ED revisits and length of hospital stay (LOHS)	SR	Adults ≥ 65 (761)	9 (1996–2015)	RCTs, PBAs	Canada, Australia, Denmark, Scotland, USA	Cochrane, Medline, CINAHL, Embase, Scopus, WOS	EBL critical appraisal tool (yes/total > 75% = valid), RevMan ROBIS tool 1 (yes) 64%, 1 (yes) 55%, 7 RCTs details: in table	Narrative synthesis & Meta-analysis
Maninchedda 2023 Italy [72]	To identify the characteristics of the problem, analysing the proposed strategies aimed at improving patient flow, delay in services provided and overcrowding of emergency departments	SR	Patients ≥ 13 Not reported	19 (2012–2021)	Descriptive study, Obs,	Belgium, Brazil, USA, Canada, China, Hong Kong, Israel, South Africa, UK, Taiwan, Turkey	PubMed, Scopus, WOS	Not appraised	Narrative synthesis
Manning 2023 Australia [29]	To uncover the challenges related to patient flow from a whole public hospital perspective and identify strategies to overcome these challenges	SR	N/A	24 (2015–2020)	Quantitative, Qualitative, Mixed method, SR	USA, UK, England, France, Australia, Canada, Austria, Netherlands	Emcare, PubMed	QATSDD (Score: 7 -37) 42 for qualitative & quantitative. 48 for a mixed method	Thematic analysis
Morley 2018 Australia [15]	To expand on and provide an updated critical analysis of the findings of peer-reviewed research studies exploring the causes or consequences of, or solutions to, ED crowding	SR	N/A	102 (2000–2018)	Cohort, RCR, B&A, RCT, TS, Obs, Mixed methods, Field study, nRCT, Cross-sectional, Modelling, Observational registry,	Singapore, UK, USA, Australia, Finland, Korea, Canada, New Zealand, Holland, Taiwan, Belgium, China, Sweden	Medline, CINAHL, EMBASE, WOS	SIGN 59% acceptable quality, 7% high quality, 34% low quality	Narrative synthesis
Ortíz-Barrios 2020 Australia [73]	Identifying approaches to support process improvement in emergency departments	SR	N/A	203 (1993–2019)	Not reported	Not reported	WOS, IEEE, Scopus, PubMed, Google Scholar, ACM Digital Library, Science Direct	Not appraised	Narrative review
Pearce 2023 Canada [39]	To synthesise the current literature of the causes, harms, and measures of crowding in emergency departments around the world	UR	N/A	13 (744 studies included in those reviews) (1980–2012)	SR	Canada, USA, Brazil, Australia, Iran, Saudi Arabia, New Zealand, Italy	MEDLINE, Embase	JBI checklist tool 5 low, 7 moderate, 1 high	Narrative review
Preston 2017 UK [74]	To systematically map interventions to identify frail and high-risk older people in the ED and interventions to manage older people in the ED and to map the outcomes of these interventions and examine whether or not there is any evidence of the impact of these interventions on Patient and health service outcomes	SMR	Frail and high risk older people and general populations of older people (aged > 65 years) (Not reported)	120 (2005–2016)	AR, Audit, B&A, Cross-sectional, Diagnostic accuracy, Feasibility study, RCR,Obs, Pilot project, RBAs, Cohort study, Comparative study, nRCTs,QE	USA, Australia, UK, Italy, Canada, Ireland, Switzerland, Netherlands, Singapore, Hong Kong, Spain, weden, France, Belgium, Germany, New Zealand, South Korea, Taiwan, Turkey	MEDLINE, EMBASE, Cochrane Library, WOS, CINAHL, Health Management Information Consortium, PROSPERO	Formal assessment not done (Bespoke assessment) Not reported	Narrative synthesis
Rasouli 2019 Iran [75]	To conduct a systematic review study concerning challenges, lessons and way outs of clinical emergencies at hospitals	SR	N/A	106 (2007–2018)	Peer-reviewed original articles	Not reported	PubMed, EMBASE	CASP, JBI Meta-Analysis of Statistics Assessment and Review Instrument No ratings reported	Narrative synthesis
Sharma 2020 UK [76]	To explore nurses' roles and their contributions to maintaining patient flow in acute hospitals through emergency departments	SR	Nurses in ED, ED patients N/A	34 (1993–2019)	Mixed studies, SR, QE, B&A, Exploratory, Ethnography, Cross-sectional, Grounded theory, AR, Descriptive RCT, CRT	USA, UK, Canada, Australia, Iran, India, Italy, Netherlands, Sweden	PubMed, CINHAL, BNI, ASSIA, SCOPUS, Google Scholar,	CASP 19 high quality, 11 moderate quality, 4 poor quality	Narrative synthesis
Shepherd 2022 UK [77]	To scope all radiographer-led discharge (RLD) literature and identify research assessing the merits of RLD and requirements to enable implementation	ScR	Radiographers ED patients with minor MSK injuries of the extremities	7 (2007–2018)	Audit, Pilot studies, Simulation modelling study, feasibility study, survey, mixed methods study	UK	MEDLINE, Embase, CINAHL, Scopus, Google Scholar, Radiography journal, Public Health England sources, Imaging and Therapy in Practice magazine, University of Exeter Repository	Not appraised	Narrative synthesis/ Descriptive-analytical approach
Voaklander 2022 Canada [78]	To describe and evaluate the effectiveness of interventions to improve the ED consultation process	SR	Patients presenting to ED Not reported for all studies	35 (2004–2021)	B&A, CBAs, RCT, Cohort TS	USA, Canada, South Korea, Singapore, Thailand, Taiwan, India, Ireland, Turkey	OVID, PubMed, EMBASE, SCOPUS, Dissertation & Theses Global, EBM Reviews/ Cochrane Library, Global Health OVID, CINAHL EBSCOhost, Google scholar, Emergency Medicine Journals	MINORS All included studies was considered poor	Narrative synthesis & meta-analysis
Zepeda-Lugo 2020 Mexico [79]	To evaluate the effects of lean healthcare (LH) interventions on inpatient care and determine whether patient flow and efficiency outcomes improve	SR	N/A	39 (2002–2019)	RCTs, CBAs, QE, Case‒control, Cohort, B&A	USA, Taiwan, Spain, UK, Saudi Arabia, Italy, India, Netherlands, Lebanon	PubMed, CINAHL, Cochrane Library, WOS, Scopus, Ebsco ProQuest, OpenGrey, Google Scholar	Cochrane's ROBINS-I 72% moderate, 28% serious risk of bias	Narrative review

*SR* Systematic reviews*, SMR* Systematic mapping review*, UR* Umbrella reviews*, LR* Literature reviews*, IR* Integrative reviews*, CR* Critical reviews*, Subs review* Substantive reviews*, RR* Rapid reviews*, ScR* Scoping reviews*, PR* Primary research*, EBR* Evidence-based reviews*, NR* Narrative reviews*, MAs* Meta-analyses, *RCR* Retrospective chart review*, CS* Case studies*, RCT* Randomised control trial*, CRT* Cluster randomised trial*, nRCTs* nonrandomised controlled trials *QE* Quasiexperimental studies*, B&A* Before & After Study*, RBAs* Retrospective before & After Study, *PBAs* Prospective before & After Study*, CBAs* Controlled before-after studies*, nCBAs* noncontrolled before-after studies*, Obs* observational studies*, ITS* Interrupted time series*, RMS* Repeated measures studies*, TS* Time series studies*, AR* Action research*, SAEM* Society of Academic Emergency Medicine*, ACEP* American College of Emergency Physicians*, AAEM* American Academy of Emergency Medicine*, CAEP* Canadian Association of Emergency Physicians*, CFHI* Canadian Foundation for Healthcare Improvement*, EPHPP* Effective Public Health Practice Project*, eNPs* emergency Nurse Practitioners*, IHI* Institute for Healthcare Improvement*, AHRQ* Agency for Healthcare Research and Quality*, ISQUA* International Society for Quality in Health Care, *HQCA* Health Quality Council of Alberta*, BCPCQC* BC Patient Safety & Quality Council*, HQSC* Health Quality & Safety Commission*, SAEM* Society for Academic Emergency Medicine*, CENTRAL* Central Registry of Controlled Trials*, MSK* Musculoskeletal*, MAIS* Multicultural Australia and Immigration Studies, *ROBIS* Risk of Bias in Systematic Reviews*, Cochrane's ROBINS-I* Risk of Bias in Nonrandomised studies of interventions*, GRADE* Grading of Recommendations Assessment*,* Development, and Evaluation*, AMSTAR* A Measurement Tool to Assess Systematic Reviews*, JBI SUMARI* JBI System for the Unified Management*,* Assessment and Review of Information, Cochrane *EPOC* Cochrane Effective Practice and Organisation of Care*, NHLBI* National Heart, Lung, and Blood, *NICE* National Institute for Health and Care Excellence*, EBL* Evidence-based librarianship*, QATSDD *Quality Assessment Tool for Studies with Diverse Design*, SIGN* Scottish Integrated Guidelines Network critical appraisal tool*, CASP* Critical Appraisal Skills Program*, MINORS* Methodological Index for Nonrandomised Studies tool

### Search characteristics: databases, countries, aims of the studies

The search dates varied across the reviews, indicating a range of timeframes for the included studies. The number of studies included in each review varied significantly, ranging from four [61] to 268 [69] studies. The majority of studies included in the analysis originated from Canada, Australia, the USA, and the UK. Among these countries, Canada had the highest number of studies, with a total of 10 [31, 36, 39, 64, 65, 68–71, 78]. The most frequently searched databases were PubMed and Medline, indicating their popularity among researchers. Additionally, grey literature sources were searched in 18 of the reviews.

The aims of the included studies can be classified into three main categories. The first category focuses on solutions for various challenges encountered in the emergency department, such as access block, consultation time, ED length of stay, ED overcrowding, nonurgent attendance in the ED, and ED boarding [13, 15, 27, 29, 31, 34, 39, 56, 58, 61, 62, 69, 72, 74, 77]. The second category involves interventions aimed at improving ED performance and utilisation. This category includes studies on throughput time, patient outcomes, provider satisfaction, older adults' experience in the ED, and ED patient care processes [7, 30, 32, 33, 35, 36, 55, 57, 59, 63–74, 76, 78, 79]. The third category encompasses studies related to challenges specifically related to patient flow [15, 29, 39].

### The results of the critical appraisal

A total of 39 reviews were assessed using the JBI Critical Appraisal Checklist for Systematic Reviews and Research Synthesis [47, 52]. The findings of the JBI Critical Appraisal Checklist for Systematic Reviews and Research Synthesis for each of the 31 reviews are summarised in Table 3. The number of criteria met varied across the reviews, with the minimum being 5 out of 11 [60] and the maximum being 11 out of 11 [57, 64, 66, 68]. Among the assessed reviews, one scoping review was determined to be of low quality [60], nine were categorised as moderate quality [13, 27, 34–36, 58, 73, 74, 77], and the remaining 27 were deemed high quality [7, 15, 29–33, 39, 43, 55–57, 59, 61–69, 71, 72, 75, 76, 78–80]. Notably, all reviews met criteria 8 and 11, which pertain to the appropriate methods used to combine studies and the recommendations for policy and/or practice supported by the reported data.

**Table 3:** Critical appraisal results for systematic reviews using the joanna briggs institute critical appraisal checklist for systematic reviews and evidence synthesis

Study	Q1	Q2	Q3	Q4	Q5	Q6	Q7	Q8	Q9	Q 10	Q 11	% Yes	Overall quality
AM 2022 [13]	Yes	Yes	Yes	Yes	U	U	U	Yes	No	Yes	Yes	64%	M
Austin 2020 [30]	Yes	Yes	Yes	Yes	Yes	Yes	Yes	Yes	No	Yes	Yes	91%	H
Beckerleg 2020 [31]	Yes	Yes	Yes	Yes	Yes	Yes	Yes	Yes	No	Yes	Yes	91%	H
Benabbas 2020 [55]	Yes	Yes	Yes	Yes	Yes	U	U	Yes	U	Yes	Yes	73%	H
Berning 2020 [32]	Yes	Yes	Yes	Yes	Yes	Yes	Yes	Yes	U	Yes	Yes	91%	H
Bittencourt 2020 [27]	Yes	Yes	Yes	Yes	Yes	No	No	Yes	U	Yes	No	64%	M
Blodgett 2021 [56]	Yes	Yes	Yes	Yes	Yes	Yes	No	Yes	U	Yes	Yes	91%	H
Boylen 2020 [57]	Yes	Yes	Yes	Yes	Yes	Yes	Yes	Yes	Yes	Yes	Yes	100%	H
Brambilla 2022 [58]	Yes	Yes	Yes	Yes	No	No	No	Yes	No	Yes	No	55%	M
Burgess 2021 [33]	Yes	Yes	Yes	Yes	Yes	Yes	Yes	Yes	U	Yes	Yes	91%	H
Cassarino 2019 [59]	Yes	Yes	Yes	Yes	Yes	Yes	No	Yes	Yes	Yes	Yes	91%	H
Clark 2022 [34]	No	Yes	Yes	Yes	N/A	N/A	No	Yes	No	Yes	Yes	55%	M
DeFreitas 2018 [7]	Yes	Yes	Yes	Yes	Yes	Yes	Yes	Yes	U	Yes	Yes	91%	H
DiLaura 2021 [35]	U	Yes	Yes	Yes	No	No	No	Yes	No	Yes	Yes	55%	M
Franklin 2022 [60]	Yes	U	Yes	U	N/A	N/A	No	Yes	No	Yes	Yes	45%	L
Gonçalves-Bradley 2018 [61]	Yes	Yes	Yes	Yes	Yes	Yes	Yes	Yes	U	Yes	Yes	91%	H
Gottlieb 2021 [62]	Yes	Yes	Yes	No	Yes	Yes	Yes	Yes	No	Yes	Yes	82%	H
Gottlieb 2021[63]	Yes	Yes	U	Yes	Yes	Yes	Yes	Yes	Yes	Yes	Yes	91%	H
Grant 2020 [64]	Yes	Yes	Yes	Yes	Yes	Yes	Yes	Yes	Yes	Yes	Yes	100%	H
Grant 2020 [65]	Yes	Yes	Yes	Yes	Yes	Yes	U	Yes	No	Yes	Yes	82%	H
Hesselink 2019 [66]	Yes	Yes	Yes	Yes	Yes	Yes	Yes	Yes	Yes	Yes	Yes	100%	H
Hong 2020 [36]	Yes	Yes	Yes	Yes	No	No	No	No	No	Yes	Yes	55%	M
Hughes 2019 [67]	Yes	Yes	Yes	Yes	Yes	Yes	No	Yes	Yes	Yes	Yes	91%	H
Jeyaraman 2022 [68]	Yes	Yes	Yes	Yes	Yes	Yes	Yes	Yes	Yes	Yes	Yes	100%	H
Jeyaraman 2021 [69]	Yes	Yes	Yes	Yes	N/A	N/A	Yes	Yes	Yes	Yes	Yes	82%	H
Kirkland 2019 [70]	Yes	Yes	Yes	Yes	Yes	Yes	No	Yes	Yes	Yes	Yes	91%	H
Leduc 2021 [71]	Yes	Yes	Yes	Yes	Yes	Yes	No	Yes	U	Yes	Yes	82%	H
Malik 2018 [80]	Yes	Yes	Yes	Yes	Yes	U	U	Yes	No	Yes	Yes	73%	H
Maninchedda 2023 [72]	Yes	Yes	Yes	Yes	No	No	Yes	Yes	No	Yes	Yes	73%	H
Manning 2023 [29]	Yes	Yes	Yes	Yes	Yes	Yes	U	Yes	Yes	Yes	Yes	91%	H
Morley 2018 [15]	Yes	Yes	Yes	Yes	Yes	Yes	Yes	Yes	No	Yes	Yes	91%	H
Ortíz-Barrios 2020 [73]	No	Yes	Yes	Yes	No	U	U	Yes	No	Yes	Yes	55%	M
Pearce 2023 [39]	Yes	Yes	Yes	Yes	Yes	Yes	U	Yes	No	Yes	Yes	82%	H
Preston 2017 [74]	Yes	Yes	Yes	Yes	N/A	N/A	No	Yes	No	Yes	Yes	64%	M
Rasouli 2019 [75]	Yes	U	Yes	Yes	Yes	Yes	Yes	Yes	No	Yes	No	73%	H
Sharma 2020 [76]	Yes	Yes	Yes	Yes	Yes	U	No	Yes	No	Yes	Yes	73%	H
Shepherd 2022 [77]	Yes	Yes	Yes	Yes	N/A	N/A	No	Yes	No	Yes	Yes	64%	M
Voaklander 2022 [78]	Yes	Yes	Yes	Yes	Yes	Yes	Yes	Yes	No	Yes	Yes	91%	H
Zepeda-Lugo 2020 [79]	Yes	Yes	Yes	Yes	Yes	Yes	No	Yes	U	Yes	Yes	82%	H

Extracted papers were considered "low quality" if the results were < 50%, "moderate quality" if they fell between 50 and 69%, and paper(s) that received > 69% were considered "high quality"

Q1: Is the review question clearly and explicitly stated?

Q2: Were the inclusion criteria appropriate for the review question?

Q3: Was the search strategy appropriate?

Q4: Were the sources and resources used to search for studies adequate?

Q5: Were the criteria for appraising studies appropriate?

Q6: Was critical appraisal conducted by two or more reviewers independently?

Q7: Were there methods to minimise errors in data extraction?

Q8: Were the methods used to combine studies appropriate?

Q9: Was the likelihood of publication bias assessed?

Q10: Were recommendations for policy and/or practice supported by the reported data?

Q11: Were the specific directives for new research appropriate?

*Y* Yes, *N* No, *U* Unclear, *N/A* Nonapplicable, *L* Low, *M* Moderate, *H* High

### Patient flow interventions

Interventions are categorised and presented in Table 4. Interventions were grouped into three main categories: (a) Human Factors; (b) Management-Organisation-Policy; and (c) Infrastructure.

**Table 4:** Main categories of patient flow improvement solutions or interventions

Main Categories	Category
Human factors	Training and professional development
	Physician-directed interventions
	Nurse-directed interventions
	Staffing adjustments
	Patient education
Management-Organisation-Policy	Process improvement
	Communication and collaboration
	Accommodating the diverse needs of patients
	Community health-related interventions
Infrastructure	Buildings and structures
	Technology/Innovation

#### Human factor interventions

In detailing human factor interventions for ED patient flow improvement, all relevant interventions are listed in Table 5. The majority of intervention examples mentioned in the studies were related to the “staffing adjustments” category. “Physician-led ED triage models” were extensively discussed in nine studies [7, 15, 27, 30, 35, 55, 64, 75, 78], highlighting their significance in optimising patient flow. “Nurse-initiated requests for paramedical service or triage nurse ordering (TNO) requests” were examined in six studies [7, 15, 27, 33, 64, 69], indicating their potential impact on improving patient flow. The “modification of staffing patterns” [7, 13, 31, 72, 75] and the “exploration of motivation and payment models” [4, 36, 64, 65, 69, 75] were addressed in five studies. In addition, training for healthcare workers received attention in four studies [29, 35, 72, 78]. It was observed that most interventions focused on the "within-ED" phase solutions, involving actions taken while patients were in the ED. While fewer interventions were identified for the “Post-ED” phase, which involves the patient's journey after discharge to home or a residential care facility, a few studies also mentioned interventions focusing on the “Pre-ED” phase, occurring before patients arrive at the ED. Education for staff in long-term care facilities, the integration of advanced nursing care within these facilities, the implementation of financial disincentives for nonemergency presentations referred by primary health care clinics, patient education through printed materials or personal contact, public education campaigns on the proper use of emergency departments, and family education are examples of interventions outside the hospital context. Overall, it was observed that most interventions within this category had mixed outcomes or conflicting evidence.

**Table 5:** Human factor interventions for patient flow improvement

Main Category: Human factors Interventions		Phases				
		**Pre-ED**	**Within-ED**	**Post-ED**		
**Category**	**Subcategories/Examples**			**Ward**	**Home**	**Residential care**
**Training and professional development**	-Triage education [30]		+			
	- Training for healthcare workers [29, 35, 72, 78]		±	±		
	- Long-term care facility staff education [65]					** = **
	- Hospital education to increase awareness of targets prior to implementation [15]		+	+		
	- Creating a supportive work environment to facilitate role development [77]		+	+		
	- Creation of new dedicated professional Figs. [72]		+	+		
**Physician-directed interventions**	- GP integration in ED for nonurgent care [27, 30, 61, 62, 69]		±			
	- Physician-led ED triage models [7, 15, 27, 30, 35, 55, 64, 75, 78]		±			
	- GP Onsite Availability (Next to ED) [30, 69]		+			
	- Dedicated neurologist in ED [64]		+			
	- Geriatrician embedded within the ED [66]		+			
**Nurse-directed interventions**	- Nurse-led triage service [7, 30, 76]		±			
	- ED nurse practitioner employment [27, 66]		±			
	- Qualified nurse for assessment, diagnosis, and treatment [7, 15, 76]		±			
	- Advanced practice nurses (clinical nurse specialist, certified registered nurse anaesthetists, clinical initiatives nurse) [7, 15, 76]		±			
	- Nurse-initiated request for paramedical service/- Triage nurse ordering (TNO) requests [7, 15, 27, 33, 64, 69]		±			
	- Integration of advanced nursing care in long-term care facilities [71]					+
	- Implementation of ED ambulance offload nurse role [66]		+			
**Staffing Adjustments**	- Changing staffing [7, 39, 72]		±			
	**-** Increasing the numbers of staff [7, 13, 15, 75]		±			
	- Modification of staffing patterns (staff types or mix) [7, 13, 31, 72, 75]		±			
	- Relocating doctors and nurses already assigned to triage in the rapid evaluation unit (RAU) [72]		+			
	- Optimised Staff Responsibilities [7, 13, 75]		±			
	- Interventions relating to Physiotherapy Roles in ED [30]		NR			
	- Interventions relating to Pharmacy Roles in ED [30]		+			
	- Dedicated ED radiology staff [7]		+			
	- Motivation, Payment models and strategies (Physician Transition to Fee-For-Service Payment, Resident health status Medicare incentives, financial incentives for PCPs and GPs) [36, 64, 65, 69, 75]		±			
	- Implementing financial disincentives for nonemergency presentations, as referred by primary health care clinics [15]	+				
	- Introduction of a team of full-time emergency medicine doctors in the ED [35]		±			
	- Scribes [7, 63, 64]		±	±		
**Patient education**	- Patient education by means of printed material or personal contact [13]	NR	NR			
	- Public education campaigns on proper use of ED [15]	+			+	
	- Family education [13]					NR

Outcomes of interventions: ( +): Positive outcome; (-): Negative outcome; ( ±): Mixed outcome/conflicting evidence; ( =): Nonsignificant outcome/no difference; (*NR*) Not reported/limited evidence

#### Management-organisation-policy interventions

Several key interventions within the main category of management-organisation-policy interventions for patient flow improvement were prominently mentioned in the included studies (Table 6). The majority of intervention examples mentioned in the studies were related to the "structural reorganisation/operational changes" subcategory of the "process improvement" category. The most frequent intervention example was "care transitions and discharge management" for timely patient handover and discharge processes, which was extensively discussed in seven studies [29, 30, 32, 34, 67, 75, 76]. "Fast-track services" for streaming or split-flow processes of nonemergency cases [7, 15, 34, 64, 69, 73] and "team composition interventions" [30, 32, 60, 65, 69, 74] were examined in six studies. It was observed that most interventions focused on the "within-ED" phase and the "ward departure" phase of the "posted" phase, involving actions taken while patients were in the ED or ward. Fewer interventions were identified for the "post-ED" phase, which involves the patient's journey after discharge to home. Overall, most interventions within this category had mixed or nonsignificant outcomes. Some interventions related to residential care facilities and home departure, including "on-site primary and acute treatment for specific conditions in long-term care facilities" [65, 71], "Implementation of the Interventions to Reduce Acute Care Transfers (INTERACT) of long-term care patients" [71], "implementation of extended care paramedics in long-term care centres" [71], "providing long-term care facilities" [34], and "home-based healthcare optimisation" [34, 74], had positive outcomes.

**Table 6:** Management-organisation-policy interventions for patient flow improvement

Main Category: Management-organisation-policy interventions		Phases				
		**Pre-ED**	**Within-ED** **Ward**	**Post-ED**		
**Category**	**Subcategories/Examples**			**Ward**	**Home**	**Residential care**
**Process improvement**	**Triage Process and protocol**					
	- Triage protocol to guide ambulance clinician's decision-making [56]	±				
	- Triaged on scene [56]	NR				
	**-** Prehospital or ED based diversion strategy [56, 70]	** = **	** = **			
	**-** Low-Acuity Patient management at triage/Management of low priority tag [13, 15, 56, 69, 72]	±	±			
	- Paramedics' accurate patient triage in on-scene triage [56, 67]	+				
	-Tailored Care Pathways through Screening [30, 78]		NR			
	- Streamlined consultation-to-decision process/Restructuring the consultation process [13, 15, 31, 78]		±			
	- Observation unit interventions [30]		NR			
	- Staggering of Elective Surgeries [34]			+		
	**Structural reorganisation/Operational Changes**					
	**-** Overcapacity protocols [7, 15, 27, 31, 75]		±	±		
	**-** Extended operating hours (after-hours primary care and ED utilisation) [7, 36, 69, 75]	±	±	±	±	
	**-** System-wide interventions/whole systems approach [7, 29]	+	+	+		
	**-** Enhanced ED workflow (process) redesign [7, 30, 73]		NR			
	- Implementation of resources, capacity, and demand Strategies for improvement [29, 75]		NR			
**Process improvement**	- Additional support from hospital leaders and specialists provided to the ED during crowded periods [15, 75]		+			
	- Standardise ED efficiency measures [35]		NR			
	- Application of queuing theory to optimise patient flow [73]		+			
	- Lean approach for ED process redesign [34, 35, 73, 75, 79]		±	±		
	- Application of six sigma for improving the patient flow [15, 75, 79]		±	±		
	- Implementing contingency strategy [75]		NR			
	- Application of the Plan-Do-Check-Act (PDCA) or Plan, Do, Study, Act (PDSA) cycle for solving LOS and discharge problem [29, 73]		+	+		
	- Data-driven management and implementation of a data-driven stat lab [29, 72, 75]		NR	NR		
	- Standardisation of the admission process [15, 31, 75]		+			
	- Implementation nationally mandated, timed patient disposition targets and guidelines [7, 31, 75]		NR			
	- Bedside registration [7, 15, 72]		+			
	- Interventions to bypass ED consultations with direct admission [78]		+			
	- Capacity Command Centers (CCCs) for patient flow management [60]		+	+		
	- Expanded Point of Care Testing [7, 30, 34, 35, 64]		±			
	- Prioritising laboratory tests/Shorter turnaround-times for laboratory tests [7, 29]		+	+		
	- Quality improvement program with feedback [65, 73]					+
**Process improvement**	- Care transitions (handover processes) and discharge management/Timely patient handover and discharge processes [29, 30, 32, 34, 67, 75, 76]		NR	+	+	NR
	- Identifying discharges, the day before [29]			NR		
	- Lateral transfers and flexible bed allocation [34]		±	±		
	- Investing in primary care [72]	+				
	- Fast-Track Services/Streaming or Split-flow processes (for nonemergency cases) [7, 15, 34, 64, 69, 73]		±			
	- Re-evaluating all patients staying in hospital for ≥ 14 days to facilitate their discharge [72]			+		
	- Monitoring the ICU and cardiac telemetry census [15]			+		
	- Minimising delays for patients being admitted [13]			+		
**Communication and collaboration**	**Care Coordination and Management**					
	- Implementation of coordinators/care coordination [7, 75, 76]	±	±	±		
	- Formation of huddles and bed management meetings/bed management and bed allocation [15, 29, 75, 76]		+	+		
	- Refined patient assignment and referral [30]		NR			
	-On-site primary and acute treatment for specific conditions in long-term care facilities [65, 71]					+
	- Implementation of a surgical specialised care team [78]		+			
	- Team composition interventions [30, 32, 60, 65, 69, 74]		±			±
	- Transfer documentation from long-term care to ED and vice versa [65]		** = **			** = **
	- Physician‒nurse triage teams/PHCPs (GPs, NP and nurses with increased authority in ED triage) [27, 29, 64, 68, 69]		+			
**Communication and collaboration**	**Integrated/collaborative care**					
	- Mental health team collocation models [34, 64, 70]			±		
	- Early Interdisciplinary Assessment and Intervention in ED [15, 30, 59, 64, 74]		±			
**Accommodating the diverse needs of patients**	- Bridge care for older adults occurring before and after ED discharge [67]	NR				NR
	- Geriatric focused nurse assessment and intervention in the ED [74, 80]		±			
	- Integration of risk screening and comprehensive geriatric assessment into primary care [80]	±				
	- "No wait" policy for older adults (immediate room placement) [32]		+			
	- High-risk elderly patient identification (readmission prevention) [32]		NR			NR
	- Acute care emergency surgery service provision (ACCESS) [75]		+	+		
	- Geriatric ED patient liaison [32, 66, 74]		NR			NR
	- Geriatric ED unit [32, 66, 67, 74]		±			±
	- Implementation of end-of-life or palliative care services [71]					+
	- Geriatric acute care unit [32, 74]		±			
	- Aged Care Pharmacist Intervention [32, 67, 68, 74] (Patient education, medication reconciliation, and referrals)		±	±		±
	- Creating a frail-friendly environment in the ED [80]		NR			
	- Implementation of a Stroke Discharge Nurse Navigator Program [76]			+		
	- Implementing a Radiographer-Led Discharge (RLD) Program for minor injuries [77]		+			
	- Implementation of the Interventions to Reduce Acute Care Transfers (INTERACT) of long-term care patients [71]					+
	- Implementation of Extended Care Paramedics in long-term care centres [71]					+
	- Availability of surgeons to provide nontraumatic surgical consults [78]		+			
	- Specialised observation units [30]		NR			
	- Patient-centred discharge coordination [32]		+			
	- Volunteer-led patient support and engagement [32]		NR			
	- ED hearing loss screening and assistive listening device provision [32]		+			
	- Colocated psychiatry liaison personnel and spaces [30, 78]		±			
	- Implementing Prognostic and diagnostic tools to identify frailty [74]		±	±		
	- Professional Interpreters in ED (Language Support) [57, 75]		+			
**Community health-related interventions**	- Increases in community-based healthcare capacity, accessibility and infrastructure (prehospital care, patient-centred medical home, rural health clinics) [13, 29, 34, 69, 74]	NR				
	- Home-based healthcare optimisation [34, 74]				+	
	- Free access to primary care for the uninsured [69, 75]	+			+	
	- Providing long-term care facilities [34]					+
	- Epidemiology-based interventions [34]				NR	

Outcomes of interventions: ( +): Positive outcome; (-): Negative outcome; ( ±): Mixed outcome/conflicting evidence; ( =): Nonsignificant outcome/no difference; (*NR*): Not reported/limited evidence

#### Infrastructure interventions

The most frequently mentioned interventions within the infrastructure category included the “implementation of simulation and predictive models or the utilisation of predictive tools” [29, 30, 35, 72, 73, 75], as well as “electronic board tracking or electronic patient tracking systems” [7, 15, 34, 35, 64, 75] (Table 7). These interventions received more attention in six studies, primarily addressing the "within-ED" phases, as well as the "ward departure" phase during “post-ED” processes. However, fewer interventions were identified for the "output" phase, which involves the patient's journey after discharge to either home or residential care facilities. Overall, the outcomes of most interventions within the technology/innovation category were mixed or nonsignificant. Some specific examples, such as “the use of instant messaging for real-time communication between ED physicians and consultants” [78], “the implementation of strategies to reduce acute care transfers for long-term care patients” [71], and “the utilisation of capacity alert escalation calls” [75], yielded positive results.

**Table 7:** Infrastructure interventions for patient flow improvement

Main Category: Physical Infrastructure Interventions		Phases				
		**Pre-ED**	**Within-ED** **Ward**	**Post-ED**		
**Generic Category**	**Subcategories/Examples**			**Ward**	**Home**	**Residential care**
**Buildings and structures**	**Buildings**					
	- Acute Medical Units (AMU) for community inpatient care [27]	+				
	- Acute care unit within ED to receive patients who need inpatient services from the ED [30]		+	+		
	- Establishing the adjacent/colocated primary care clinic for lower acuity patients [69]	NR			NR	
	- Rapid assessment zones for expedited patient evaluation and treatment [7, 13, 27, 30]		±			
	- Short Stay Units (SSUs) for streamlined ED patient care [7, 13, 27, 30]		±	±		
	- Alternative Free Standing Emergency Departments (FSEDs) [58]	NR				
	- Opening additional EDs [75]		NR			
	- Implementing GP-led walk-in centres and colocating GPs [57]	** = **			** = **	
	**Physical structures**					
	- Increasing the numbers of beds or freeing beds [13, 34, 72, 75]	±	±			
	- Reclining hospital chair [32]		+			
	- Increasing the size of EDs [13, 34]		** = **			
	- Hallway emergency bed policy (reorganisation of internal spaces for first patient evaluation using hallway beds/chairs) [72, 75]		±			
	- Provision of patient lounges to support admission-discharge patient flow (Transit lounges) [34]		+	+		
	- Allocating financial resources for patient flow enhancement [34]	NR	NR	NR	NR	
**Technology/Innovation**	**Telehealth**					
	- Telemedicine triage/Online ‘pre-ED’ triaging [13, 64]	±				
	- Telehealth care service/virtual care/visit systems [13, 35, 65, 71]	±			±	
**Technology/Innovation**	**Information Technology (IT)**					
	- Clinical Decision Support Systems (CDSS) [29, 30, 65, 73]		NR			NR
	- Web-based dashboards and reporting applications to provide real-time information and monitor patient flow [29, 73, 75]		NR	NR		
	- Implementing community-based Regional Transfer Network System (RTNS) [75]		+	+		
	- Using capacity alert escalation call [75]		+	+		
	- Mobile Devices [30]		NR			
	- Computerised Provider Order Entry (CPOE) [7, 35, 64]		±	±		
	- Integrated ED Information System [35]		NR			
	- Implementation of simulation and predictive models/Discrete event simulation (DES)/predictive tool [29, 30, 35, 72, 73, 75]		NR	NR		
	- Electronic board tracking/electronic patient tracking systems/electronic Blockage System (EBS) [7, 15, 34, 35, 64, 75]		+	+		
	- Leverage machine algorithm learning [29, 73]		NR	NR		
	- AI-powered automatic patient‒physician assignment [64]		NR			
	- Implementing a random monitoring system of the ambulance block [72]	NR	NR			
	- Telephone consultations [65]					NR
	- SMS reminder to consultant/residents about consultation delays [15, 31, 78]		NR			
	- Use of instant messaging (e.g., WhatsApp) for real-time communication between ED physicians and consultants [78]		+			
	- Electronic Health Records (EHR) Access [30, 60, 64, 72]		NR			

** + **: Outcomes of interventions: ( +): Positive outcome; (-): Negative outcome; ( ±): Mixed outcome/Conflicting evidence; ( =): Nonsignificant outcome/No difference

(*NR*) Not reported/Limited evidence

### Outcomes of patient flow improvement solutions

The outcomes of patient flow improvement solutions are classified in Table 8.

**Table 8:** Outcomes of patient flow improvement solutions

Main category [31]	Category	Resource No	QAIMS
**Proportion-related outcomes**	Reduction of ED-LOS/Hospital LOS	[7, 15, 27, 31, 34, 35, 39, 55, 60–64, 66, 68, 69, 71–74, 76–79]	QAIM3
	Reduction of Patients left without being seen (LWBS)/Did not wait (DNW)	[15, 27, 34, 35, 60, 64, 66, 68, 69, 72, 73]	QAIM1
	Reduction of patients leaving against medical advice (LAMA)	[68, 72]	QAIM1
	Access block reduction	[13, 15]	QAIM1, QAIM3
	Meeting NEAT targets	[15, 73]	QAIM1, QAIM4
	Manageable ED occupancy level	[39, 66, 74]	QAIM3
	Decrease in Turnover Time (TOT)	[70]	QAIM3
	Decrease in Turnaround Time (TAT)	[70]	QAIM3
	Decrease in hospitalisations	[27, 74]	QAIM3
	Decrease in admission rates	[33, 59, 65, 67, 71, 74]	QAIM3
	Decrease in weekend ED attendances	[15]	QAIM2
	Decrease in ED visits (ambulance admissions & self-referrals)	[15, 36, 68–71, 74, 76, 80]	QAIM3
	Reduction In the number of Non-Urgent/Semi-Urgent/frequent users	[36, 73, 74]	QAIM2
	Improvement of Discharge Rates	[34, 74]	QAIM3
	Improvement of on-time starts (OTS)	[79]	QAIM3
	Decrease in Readmission/Revisit Rates/Relapse	[34, 39, 61, 65–67, 71, 74, 79, 80]	QAIM3
	Decrease in waiting time	[7, 39, 66, 73, 74, 76]	QAIM3
	Reduction in ED transfer rate	[65, 73]	QAIM3
	Decrease in Triage to ED Room/Bed Placement Time	[7, 66, 68, 76]	QAIM3
	Decrease in door to physician time/time to Physician Initial Assessment	[7, 60, 62, 68, 69, 72, 76, 78]	QAIM3
	Decrease in time to initiation of diagnostic testing	[72, 74]	QAIM3
	Decrease in consult response time	[78]	QAIM3
	Decrease in consultation to decision time	[27, 31, 66, 78]	QAIM1, QAIM1
	Proportion of patients consulted	[78]	QAIM4
	Decrease in physician to disposition decision time/ED workup time	[7, 69]	QAIM3
	Reduced patient lead-time from registration to discharge	[73, 77, 79]	QAIM3
	Decrease in Time-to-Treatment	[7, 15, 33, 61, 66, 72]	QAIM3
	Decrease in ED boarding hours or time or count	[15, 34, 60, 66, 79]	QAIM3
	Increase in patients transferred to inpatient bed	[15]	QAIM1, QAIM4
	Number of patients diverted to primary care	[69, 70]	QAIM3
**Cost-related outcomes**	Lower costs	[60, 61, 64, 65, 68–74, 78, 79]	QAIM3
	Resource utilisation	[74]	QAIM3
**Process related outcomes**	Reducing overcrowding	[13, 27, 39, 73, 76]	QAIM1
	Enhanced throughput efficiency	[34]	QAIM3
	Streamlined door-to-physician process	[35]	QAIM3
	Enhanced referrals to community services	[59, 74]	QAIM2
	Parental hospital visit satisfaction –	[57]	QAIM2
	Reduction in hours of ambulance bypass/diversion	[15, 39, 66]	QAIM3
	Enhanced patient access to ED	[34]	QAIM3
	Decrease in the Emergency Department Work Index (EDWIN) score	[39]	QAIM3
	Reducing ED utilisation	[36, 69, 73]	QAIM3
**Patient or provider related outcomes**	Improve the patient experience	[15, 32, 35, 39, 55, 57, 59, 63, 67–69, 72, 73, 76, 77, 79, 81]	QAIM1
	Improved clinical experiences	[29, 76, 79]	QAIM4
	Decrease in serious adverse event (e.g., mortality, ICU admission)	[15, 35, 39, 70–72]	QAIM2
	Enhanced patient safety	[59, 69]	QAIM1
	Improved health-related quality of care	[59]	QAIM1
	Improvement in Patient's quality of life	[70]	QAIM1
	Adherence to treatment	[57]	QAIM1, QAIM2
	Decrease in potentially avoidable diagnostic tests and treatments	[72]	QAIM3
	Reduction in medication errors	[39, 57, 74, 77]	QAIM1, QAIM4
	Symptom relief	[33]	QAIM1
	Reduced ED staff stress level	[15, 66]	QAIM4
	Satisfaction of staff	[15, 59, 63, 72, 79]	QAIM4

AIMs: QAIM1: Improve the patient experience of care

QAIM2: Improved population health

QAIM3: Reduced cost and improved efficiency

QAIM4: Enhance the work-life balance and satisfaction of healthcare providers

Several outcomes or aims of patient flow improvement solutions were frequently mentioned in the included studies, with "reduction of ED-LOS/hospital LOS" discussed in 24 studies [7, 15, 27, 31, 34, 35, 39, 55, 60–64, 66, 68, 69, 71–74, 76–79], "improving the patient experience" in 17 studies [15, 32, 35, 39, 55, 57, 59, 63, 67–69, 72, 73, 76, 77, 79, 81], “lowering costs” in 13 studies [60, 61, 64, 65, 68–74, 78, 79], “reducing patients left without being seen or those who did not wait (LWBS/DNW)” in 11 studies [15, 27, 34, 35, 60, 64, 66, 68, 69, 72, 73], and “decreasing readmission or revisit rates” [34, 39, 61, 65–67, 71, 74, 79, 80] and “reducing ED visits” [15, 36, 68–71, 74, 76, 80] in 10 and 9 studies, respectively, reflecting the multifaceted nature of these solutions and highlighting key areas of focus in optimising healthcare delivery.

The aims to improve the patient experience of care, reduce LWBS/DNW/LAMA, decrease in consultation to decision time, and reduce overcrowding are addressed under QAIM1, while QAIM2 focuses on improving population health, enhancing referrals to community services and parental hospital visit satisfaction, decreasing potentially avoidable diagnostic tests and treatments, and reducing the number of nonurgent, semiurgent, and frequent users. QAIM3 is mapped to reducing costs, ED-LOS and hospital LOS, optimising resource allocation with better clinical outcomes, and decreasing admission rates, ED visits, waiting time, door-to-physician time, and ED boarding hours. In addition, QAIM4 encompasses improving the clinical experiences and satisfaction of staff and reducing ED staff stress levels.

### Patient flow challenges across the healthcare system

The findings on root causes of ED patient flow challenges presented in Table 9 were categorised based on the population-capacity-process (PCP) model.

**Table 9:** Root causes of patient flow challenges and their outcomes across the healthcare system

Category	Subcategory	Outcomes of challenges No
Population Patients & Providers)	Demand fluctuations (changes such as seasonal increases in demand, and unanticipated events [56]	4,5
	Patient's characteristics/Patient-related factors (extremes of age, critically ill, social determinants of health and …) [39, 55, 75, 76]	1,3,5,7,9,10,11,16
	Acuity mix of the patients in the ED [15, 36, 55, 61, 68, 72, 73]	1, 3, 4, 5,7,8,9,10,11,12
	Rising demand for ED visits and hospitalisation due to aging population [15, 32, 66, 72, 74]	2, 5,11
	Increase of the poor population with consequent difficulty to face health costs [72]	5,11
	Mismanagement of treatable diseases at home [72]	5,11
	Language differences [57]	5
	High ED staff stress level and burnout [57, 66, 76]	9,13
	Excessive workloads [39, 75]	4,5,6,13,15
	High staff turnover [39]	13
	Lack of awareness of systems and processes particularly among temporary staff [76]	5,8
	Insufficient training of professionals practicing in the ED [56, 66, 76]	5
Capacity	Limited bed availability [13, 27, 34, 72, 73, 75, 76, 79]	4,5,6,7,8,9,11,12,16
	Physical or architectural limitations in the ED [7, 72]	11
	Mismatch between capacity and demand [29, 73]	4,5,7,8,12
	High number of patients in the waiting room [39, 75]	5,7,8,12,13,15
	High percentage of beds occupied by boarders [39]	7,13,15
	Occupancy rate of the ED and hospital [39, 55]	1, 3,7,9,10,13,15
	Rising burden of chronic disease [15, 72]	11,15
	Inappropriate ED utilisation/visits [39, 69, 70, 75, 76]	6,9,13,15
	Rising readmissions [75]	4,5,6,9,15
	Shortage of hospital discharge rooms [72]	5,11
	Limited human resources/Health care understaffing [7, 15, 27, 39, 55, 58, 72, 73, 75, 76]	1, 3, 5,7,8, 9,10,11,12
	Unavailability of Healthcare Assistants [76]	8
	EMS traffic/volume [68]	4,5,6
	Lack of social services to facilitate difficult patients’ discharge	5,8,11
	Number of admissions [38, 56, 70]	1,3, 4,7,9,10,13,15
	Reduced health funding [72]	5
	Increased inpatient length of stay (IPLOS) [15]	5,6,11
	Limited access to diagnostic services in community [15]	5,8
	The high daily census of inpatient critical care and cardiac telemetry units [15]	7
	Insufficient availability of beds in community-based care settings [76]	2,4,5
	Time and day variations in patient flow[55]	1, 3, 7, 9, 10
Process	Challenges with diverting low acuity patients from ambulances to alternative care sites [55, 72, 73]	5,7,8,12
	Insufficient communication and poor collaboration between teams [29]	6,15
	Limited primary care access [15, 39, 72]	5,11,14
	Failure to identify available beds and fragmented bed management process [29, 72]	5,11
	Exit block, delayed discharge, and delayed disposition decisions [15, 29, 34, 39, 72, 75]	5,6,7,8,9,11,15,16
	Boarding time [39, 75]	5,7,9,15
	Demand for diagnostic tests and imaging studies/delays in receiving test results [15, 39, 55, 75]	1, 3,5,7,8, 9,10,11,13,15
	Inadequate integration of ED facilities with imaging and diagnostic departments, on-call specialists, and extended medical services [58, 72, 75]	7,8,5,11
	Lack of health care network integration [27]	6,15
	Ineffective transitions of care/Referral patterns [55]	3, 7, 9, 10
	Prolonged trainee assessment and review time/presence of junior medical staff in ED [15, 31, 76]	4,5
	Collaboration lack between health personnel [72]	5,8,11
	The reluctance of hospital staff to admit patients from ED [75]	5,7,11,15
	Inability of staff to adhere to guideline-recommended treatment [15]	5
	Difficulties and issues encountered during the triage process [27, 60, 72]	5,7,8,12
	Difficulties in accessing urgent healthcare service [72]	5,8,11
	Ineffectiveness of Interventions targeting frequent ED users [72]	5,11
	Limitations on nurses' authority to initiate certain treatments [33]	8
	Low effectiveness of basic care services [27]	5,6
	Lack of seasonal disease prophylaxis [72]	5,11
	System complexity" or "complexity of public hospitals [29]	15
	Variations in local emergency medicine/Differences in emergency practice [36]	7,4
	Wrong diagnosis [75]	5,7,9,11,16
	Lack of Integration between EDs and Inpatient Services [13]	4, 6
	Consultation delays [15, 31, 39, 72, 75]	5,8,11,13,15

Outcomes of barriers/challenges No:

1. Adverse outcomes upon leaving the ED

2. Increased LOS for older adults

3. Public relations risk for healthcare systems

4. Hospital overcapacity

5. ED Crowding

6. Access block or nonflow

7. Prolonged ED-LOS

8. Extended waiting time, Delayed progression of care

9. Significant financial risk for healthcare systems/increased costs of healthcare

10. Significant medicolegal risk for healthcare systems

11. Patient dissatisfaction

12. LWBS: Patients left without being seen

13. Decreased ED quality of care (QoC)

14. Increase in mental health and addiction presentations

15. Poor patient throughput

16. Increase in adverse effects and deaths

Among the factors related to the population, root causes frequently identified were “the acuity mix of patients in the ED” [15, 36, 55, 61, 68, 72, 73], “rising demand for ED visits and hospitalisation due to an ageing population” [15, 33, 71, 77, 78], and “patient characteristics” [38, 58, 59, 67]. The most common capacity challenges included “limited human resources” [7, 15, 28, 38, 57–59, 67, 68, 71], “limited bed availability” [13, 27, 34, 72, 73, 75, 76, 79], and “inappropriate ED utilisation or visits” [39, 69, 70, 75, 76]. Process-related challenges encompassed issues with communication, test results, primary care access, transitions of care, and low-acuity patients. Notable challenges reported were “exit block and delayed transitions of care” [15, 29, 34, 39, 72, 75], “consultation delays” [15, 31, 39, 72, 75], “delays in demanding and receiving diagnostic tests and imaging studies” [15, 39, 55, 75], “limited primary care access” [15, 38, 71], and “difficulties in diverting low acuity patients from ambulances to alternative care sites” [55, 72, 73].

The challenges presented a wide range of outcomes, encompassing adverse patient outcomes, extended length of stay, ED crowding, financial risks for healthcare systems, and patient dissatisfaction. The study identified ED crowding (*N* = 41 root causes), patient dissatisfaction (*N* = 25 root causes), prolonged ED-LOS (*N* = 21 root causes), and extended waiting time (*N* = 1 root cause) as the most frequent outcomes resulting from the identified root causes of patient flow challenges.

## Discussion

### Patient flow interventions or solutions

Categorising interventions into human factors, management-organisation-policy, and infrastructure provides a comprehensive understanding of evidence-based strategies to improve patient flow. Additionally, Rasouli et al. broadly categorised approaches or solutions to reduce or prevent ED overcrowding into organisation- or management-level interventions and operation-level interventions [75]. Furthermore, Freitas et al. categorised interventions aimed at improving patient flow and reducing overcrowding into several groups, including diagnostic services, assessment/short stay units, nurse-directed interventions, physician-directed interventions, administrative/organisational interventions, and miscellaneous interventions [7]. Moreover, an overview by Conneely highlighted various interventions, such as gerontologically informed nursing assessment, comprehensive geriatric assessment, ED community transitional strategies, ED-based interventions, and single/multistrategy interventions initiated in the ED [28].

### Human factor interventions

In the category of human factor interventions, various interventions have been identified. Similarly, various training and professional development interventions were identified in the studies [30, 65]. These interventions encompassed training sessions on a new rapid assessment and disposition process, brief orientation to the new process, education to increase awareness of national targets, an education day with a focus on specific areas of improvement, training for nurses in coordinating communications, pain management, and triage [24, 82–85]. Furthermore, Anantharaman et al. found that public education on the proper use of the emergency department can be effective in the short term but may not have a lasting impact. To ensure sustained desired outcomes beyond the education period, additional strategies or interventions may be needed [15, 86]. Physicians working alone or alongside nurses in triage allowed for prompt diagnostic procedures and treatments, leading to reduced length of stay and waiting time per patient [27]. However, some argue that team triage lacks clear advantages and sufficient evidence regarding its benefits [28, 87, 88]. These interventions primarily focus on "within-ED" solutions to enhance the flow and efficiency of the ED.. However, there is a deficiency in interventions addressing the "post-ED" phase to enhance ED patient flow. This phase involves the patient's journey after discharge, including home departure or residential care departure. Additionally, there is a gap in interventions for the "pre-ED" phase, which includes supporting patients at home and redirecting them to more appropriate types of care, such as primary care and other urgent ambulatory care services. Similarly, the findings of Gettel et al. revealed that ED-to-community care transitions often lack effective care coordination and communication, especially for older adults with cognitive impairment [89].

#### Management-organisation-policy interventions

Notably, structural reorganisation and operational changes have also been frequently mentioned within the management-organisation-policy category, with a focus on care transitions, discharge management, and fast-track services. Aligned with the findings of Ortíz-Barrios et al., various process improvement methodologies have been employed to address crucial issues in emergency departments, including overcrowding, prolonged waiting time, extended length of stay, excessive patient flow time, and high rates of LWBS [73]. It is crucial for future efforts to involve ED administrators, researchers, and stakeholders in designing comprehensive strategies utilising operations research (OR) methods to enhance ED performance and address these specific challenges. Additionally, these interventions predominantly concentrate on solutions in the "within-ED" and "ward departure" phases, and interventions addressing the "post-ED" phase, especially the home departure phase (from either ED or the inpatient wards), to improve ED patient flow are relatively sparse. Alharbi et al. similarly discovered that efforts to reduce inpatient long stays were impacted by various constraints, which included the challenge of meeting the postdischarge needs of specific patient populations. For instance, ventilated patients faced difficulties due to the absence of specialised long-term care units capable of accommodating them and the unavailability of home services resulting from a shortage of trained and dedicated healthcare workers in their area [90].

#### Infrastructure interventions

The included studies particularly highlighted telehealth and information technology (IT) interventions for improving ED patient flow. The implementation of simulation and predictive models or the utilisation of predictive tools, as well as electronic board tracking or electronic patient tracking systems, were among the most frequently mentioned interventions. The main focus of these interventions to enhance ED patient flow was on solutions “within the ED” and ward departure. However, fewer interventions were identified for the post-ED phase, which involves the patient's journey after leaving the ED. Overall, the outcomes of most interventions within the Technology/Innovation category were mixed or nonsignificant. Based on available evidence, various technologies, such as nurse call lines, on-demand telehealth visits, tele-triage, and paramedic-driven mobile response programs, were identified as valuable tools for screening patients before their arrival at the emergency department, aiming to mitigate ED overcrowding [91, 92]. Additionally, telemedicine tools such as remote patient monitoring and virtual visits have been employed in home hospital settings, while virtual observation units facilitate early discharge from hospitals or emergency departments, enabling patients to transition to home care [79, 93, 94].

#### The outcomes of patient flow improvement solutions

The overall results of the study indicate a mixed picture in terms of the outcomes of interventions aimed at improving patient flow. While some interventions showed positive outcomes, such as specific interventions targeting residential care facilities and home departure, as well as certain technology-based interventions, the majority of interventions yielded mixed, conflicting, or nonsignificant outcomes. The study's results align with prior overview studies, which suggests that evidence concerning the effectiveness of interventions in ED settings and patient flow is both limited and ineffective. This is due to the heterogeneity of methods, populations, and measured outcomes, which makes it difficult to compare the results of different studies and draw firm conclusions about the effectiveness of interventions [7, 27, 28]. These findings highlight the importance of establishing and utilising a comprehensive range of meaningful outcome measures to accurately evaluate the effectiveness of interventions on patient flow.

The outcomes of ED patient flow solutions encompass a wide range of categories, in accordance with the quadruple aim framework. Other studies have similarly found that process improvement and rapid assessment implementation had a more significant impact on improving ED productivity and performance compared to renovation and facility expansion [95, 96]. By enhancing ED operational efficiency, the healthcare facility was able to handle increased patient volume while simultaneously improving the quality of care and patient satisfaction [97–99]. Remarkably, these improvements were achieved with minimal additional resources, space, or staffing [99].

### Patient flow challenges

Within the population factors, the most commonly identified root causes were the acuity mix of patients in the ED, the rising demand for ED visits and hospitalisation due to an ageing population, and patient characteristics. Capacity challenges often revolve around limited human resources, limited bed availability, and inappropriate ED utilisation or visits. Process-related challenges encompassed issues with communication, test results, primary care access, transitions of care, and low-acuity patients. In this regard, Manning's study identified five areas of challenges: teamwork, collaboration and communication; public hospitals as complex systems; timely discharge; policy, process, and decision-making; and resources, capacity, and demand [29]. Additionally, according to Morely's report, the predominant causes identified were associated with the volume and demographics of individuals seeking care at the ED, as well as the timely discharge of patients from the ED [15]. Our study also showed that these challenges resulted in various outcomes, including adverse patient outcomes, extended length of stay, ED overcrowding, financial risks for healthcare systems, and patient dissatisfaction. The most frequent outcomes from the identified root causes were ED overcrowding, patient dissatisfaction, prolonged ED-LOS, and extended waiting time. Other studies have found that ED crowding is a complex issue with multiple contributing factors. These factors can be found in the input, throughput, and output areas of the ED [13, 92, 100]. These findings highlight the complex nature of ED patient flow challenges and underscore the need for targeted interventions and system-level changes to address them effectively.

## Conclusion

The findings of this study reveal a mixed impact of interventions on patient flow. The evidence available is often of lower quality, consisting mostly of cross-sectional and noncontrolled pre- and postdesign studies. The variation in geographic areas and healthcare systems among the included studies further complicates the interpretation of results. Insufficient evidence exists to definitively support the effectiveness and safety of diversion strategies and other interventions. Many of the initiatives examined in the literature were pilot projects or quality improvement projects, lacking rigorous evaluation against comparator groups. Inconsistencies in assessment and interventions for patient flow improvement are evident, highlighting the need for standardised measures and evidence-based solutions.

Despite efforts and accumulated knowledge, the problem of ED overcrowding remains a global challenge, indicating the limited success in implementing evidence-based solutions for improving patient flow. The focus on ED interventions in the included reviews limits their usefulness for understanding interventions across the care pathway. It is crucial to utilise a comprehensive range of meaningful outcome measures to accurately assess the effectiveness of system-wide interventions and inform system changes and decision-making. The focus on speed, rather than quality or experience of care, is concerning.

Future research should focus on evaluating the effectiveness of specific interventions using consistent conceptual models and standardised measures. The potential displacement of care resulting from interventions to reduce inappropriate admissions should be explored, along with the impact of healthcare professionals delivering the interventions. Further investigation is needed on interventions combining ED care with home follow-up and different models of discharge management. Community screening to identify high-risk patients and diverting frail older individuals from ED presentations may be more effective.

The findings from this literature review suggest the following recommendations:

1. Developing patient flow interventions from the pre-ED phase to the post-ED phase to enhance patient flow in the ED: Given the identified gap that the majority of interventions primarily target the 'within-ED' phase, there is a crucial need to expand interventions for the 'post-ED' and 'pre-ED' phases. This includes strategies for smooth care transitions, effective discharge management, improved access to primary care, diversion of frail older individuals from ED presentations, and efficient transitions for patients after leaving the hospital. By comprehensively addressing these areas across the healthcare system, healthcare systems can mitigate readmission rates, enhance patient satisfaction, and optimise overall patient flow. 2. Embrace community-based care interventions to address the identified challenge of insufficient availability of beds in community-based care settings, which leads to increased length of stay (LOS) for older adults, hospital overcapacity, and ED crowding. Our review highlights the potential positive outcomes associated with community health-related interventions, such as home-based healthcare optimisation, providing free access to primary care for the uninsured, and establishing long-term care facilities. 3. Strengthen Technology and Innovation Interventions: Additional research is needed to investigate and enhance technology or innovation interventions that focus on all three phases of patient flow. While interventions within the telehealth and information technology subcategories have shown promise in improving ED patient flow specifically “within the ED” and during the “departure to the ward” phase, there is a need to expand their effectiveness to the “Post-ED” phase. This research should aim to optimise the implementation of predictive models, electronic tracking systems, and other technological solutions to enhance ED patient flow.

4. Establish Standardised Measures: We need to measure outcomes other than time. To accurately evaluate the effectiveness of interventions on patient flow, it is crucial to establish and utilise a comprehensive range of meaningful outcome measures. These measures should encompass proportion-related outcomes, cost-related outcomes, process-related outcomes, and patient- or provider-related outcomes. By adopting standardised measures, healthcare systems can effectively achieve the goals of the quadruple aim framework.

5. Address Root Causes of Patient Flow Challenges and Conduct Rigorous Research and Evaluation: To address the root causes identified from previous studies, it is essential to design interventions that specifically target these causes and evaluate their effectiveness using standardised measures. Healthcare systems should focus on implementing interventions that address factors such as population dynamics, capacity challenges, and process-related issues. Rigorous evaluation should be conducted to assess how these interventions effectively address the identified root causes and their impact on patient flow. This includes employing controlled studies with comparator groups and exploring potential displacement of care resulting from interventions. By linking interventions to root causes, utilising standardised measures, and conducting comprehensive evaluation, healthcare systems can build a robust evidence base and support evidence-based decision-making for optimising patient flow.

Overall, these recommendations emphasise the importance of implementing comprehensive, evidence-based interventions that address solutions across the entire patient flow process, including the phases before and after the ED visit. By focusing on human factors, management-organisation-policy, and infrastructure interventions.

## Limitations

The study had several limitations. First, unsuccessful interventions are unlikely to have been published, so this paper is subject to publication bias. Another limitation of the study was the limited number of primary studies included in the selected reviews that utilised a single intervention strategy. This scarcity made it challenging to draw definitive conclusions regarding the exact effective action component of the intervention strategy. Additionally, there was variability in study populations, intervention components of the solutions, and outcome measures across the primary studies of the included reviews, which limited the ability to make comprehensive and consistent conclusions. Furthermore, an inherent limitation of our approach lies in classifying the extracted outcomes based on the Quadruple Aim framework. For outcomes that were not explicitly mentioned in relation to a specific QAIM, we performed mapping based on our own judgment and interpretation to assign them to the relevant QAIM. It is essential to consider the specific context and how these outcomes contribute to overall improvements in healthcare and patient experiences. The classification may vary depending on the goals and priorities of the healthcare system.

### Supplementary Information

**Supplementary Material 1. **

**Supplementary Material 2. **

**Supplementary Material 3. **

**Supplementary Material 4. **

**Supplementary Material 5. **

## Data Availability

All data generated or analysed during this study are accessible in this published article and its supplemental information files.

## Abbreviations

ACCESS: Acute care emergency surgery service provision
CCA: Corrected covered area
CCCs: Capacity command centers
DNW: Did Not Wait
ED: Emergency Department
EDs: Emergency Departments
EDWIN: Emergency Department Work Index
GP: General practitioner
GROOVE: Graphical representation of overlap for overviews
INTERACT: Implementation of Interventions to Reduce Acute Care Transfers
JBI: Joanna Briggs Institute
LAWA: Reduction of patients leaving against medical advice
LOS: Length of stay
LWBS: Left Without Being Seen
NEAT: The National Emergency Access Target
NP: Nurse practitioner
OR: Operations Research
OTS: On-Time Starts
PCP: Primary Care Provider
PCP: Population-capacity-process
PDCA: Plan-Do-Check-Act
PDSA: Plan, Do, Study, Act
PHCPs: Primary healthcare professionals
PICo: Population, Phenomena of interest and Context
PRESS: Peer Review of Electronic Search Strategies
PROSPERO: Prospective Register of Systematic Reviews
QAIM: Quadruple Aim
QoC: Quality of Care
TAT: Turnaround Time
TNO: Triage nurse ordering
TOT: Decrease in turnover time
