# Supplementary material for: Patient flow in emergency departments: a comprehensive umbrella review of solutions and challenges across the health system
Source: BMC Health Serv Res. 2024 Mar 5;24:274. doi: 10.1186/s12913-024-10725-6 (PMC10913567; doi:10.1186/s12913-024-10725-6)
Supplement: Supplementary file 1 — Supplementary Material 1. [file 12913_2024_10725_MOESM1_ESM.docx]

**Appendix 1. The search strategies implemented for each database**

**PubMed: 220**

("Emergency Service, Hospital"[Mesh] OR "Emergency Medicine"[Mesh] OR "Emergency Nursing"[Mesh] OR "emergency medicine"[ti] OR "emergency nursing" OR "Hospital Emergency Service" OR "Hospital Emergency Services" OR "Emergency Hospital Service" OR "Emergency Hospital Services" OR "Emergency Department"[ti] OR "Emergency Departments"[ti] OR "Emergency Unit"[ti] OR "Emergency Units"[ti] OR "Emergency Ward"[ti] OR "Emergency Wards"[ti] OR "Emergency Room"[ti] OR "Emergency Rooms"[ti] OR "trauma center"[ti] OR "trauma centers"[ti] OR "trauma unit"[ti] OR "trauma units"[ti] OR (emergency[ti] AND hospital[ti]))

AND

("crowding"[MeSH Terms] OR crowd*[tiab] OR congest*[tiab] OR overcrowd*[tiab] OR gridlock*[tiab] OR queu*[tiab] OR overload*[tiab] OR "access block*"[tiab] OR "Patient flow"[tiab] OR "patient inflow" OR "Patient turnover"[tiab] OR "patient Caseload"[tiab] OR "patient Caseloads"[tiab] OR "patient throughput*"[tiab] OR "emergency department throughput"[tiab] OR "patient journey"[tiab] OR "patient inflow" OR "patient path*"[tiab] OR "patient disposition"[tiab] OR "patient dispositions"[tiab] OR bottleneck [tiab] OR bottlenecks[tiab] OR challenge[tiab] OR challenges[tiab] OR barriers[tiab] OR barrier[tiab] OR "Patient boarding"[tiab] OR delay[tiab] OR delays[tiab] OR"choke point"[tiab] OR "choke points"[tiab])

AND

("systematic reviews as topic"[MeSH Terms] OR "meta analysis as topic"[MeSH Terms] OR "Meta-Analysis" [Publication Type] OR "Systematic Review"[Publication Type] OR "systematic review*"[tiab] OR metaanal*[tiab] OR meta-anal*[tiab] OR metasyn*[tiab] OR meta-synth*[tiab] OR systematic[sb] OR systematic[ti] OR scoping[ti])

Filters: from 2018/1/1 - 3000/12/12 (current)= 220

Search: ("Emergency Service, Hospital"[Mesh] OR "Emergency Medicine"[Mesh] OR "Emergency Nursing"[Mesh] OR "emergency medicine"[ti] OR "emergency nursing" OR "Hospital Emergency Service" OR "Hospital Emergency Services" OR "Emergency Hospital Service" OR "Emergency Hospital Services" OR "Emergency Department"[ti] OR "Emergency Departments"[ti] OR "Emergency Unit"[ti] OR "Emergency Units"[ti] OR "Emergency Ward"[ti] OR "Emergency Wards"[ti] OR "Emergency Room"[ti] OR "Emergency Rooms"[ti] OR "trauma center"[ti] OR "trauma centers"[ti] OR "trauma unit"[ti] OR "trauma units"[ti] OR (emergency[ti] AND hospital[ti])) AND ("crowding"[MeSH Terms] OR crowd*[tiab] OR congest*[tiab] OR overcrowd*[tiab] OR gridlock*[tiab] OR queu*[tiab] OR overload*[tiab] OR "access block*"[tiab] OR "Patient flow"[tiab] OR "patient inflow" OR "Patient turnover"[tiab] OR "patient Caseload"[tiab] OR "patient Caseloads"[tiab] OR "patient throughput*"[tiab] OR "emergency department throughput"[tiab] OR "patient journey"[tiab] OR "patient inflow" OR "patient path*"[tiab] OR "patient disposition"[tiab] OR "patient dispositions"[tiab] OR bottleneck [tiab] OR bottlenecks[tiab] OR challenge[tiab] OR challenges[tiab] OR barriers[tiab] OR barrier[tiab] OR "Patient boarding"[tiab] OR delay[tiab] OR delays[tiab] OR"choke point"[tiab] OR "choke points"[tiab]) AND ("systematic reviews as topic"[MeSH Terms] OR "meta analysis as topic"[MeSH Terms] OR "Meta-Analysis" [Publication Type] OR "Systematic Review"[Publication Type] OR "systematic review*"[tiab] OR metaanal*[tiab] OR meta-anal*[tiab] OR metasyn*[tiab] OR meta-synth*[tiab] OR systematic[sb] OR systematic[ti] OR scoping[ti]) Filters: from 2018/1/1 - 3000/12/12= 220

**Embase=522**

(‘Emergency Service, Hospital’/exp OR ‘Emergency Medicine’/exp OR ‘Emergency Nursing’/exp OR ‘emergency medicine’:ti OR ‘emergency nursing’ OR ‘Hospital Emergency Service’ OR ‘Hospital Emergency Services’ OR ‘Emergency Hospital Service’ OR ‘Emergency Hospital Services’ OR ‘Emergency Department’:ti OR ‘Emergency Departments’:ti OR ‘Emergency Unit’:ti OR ‘Emergency Units’:ti OR ‘Emergency Ward’:ti OR ‘Emergency Wards’:ti OR ‘Emergency Room’:ti OR ‘Emergency Rooms’:ti OR ‘trauma center’:ti OR ‘trauma centers’:ti OR ‘trauma unit’:ti OR ‘trauma units’:ti OR (‘emergency treatment’:ti AND hospital:ti))

AND

(‘crowding (area)’/exp OR ‘crowding (area)’ OR crowd*:ti,ab OR congest*:ti,ab OR overcrowd*:ti,ab OR gridlock*:ti,ab OR queu*:ti,ab OR overload*:ti,ab OR ‘access block*’:ti,ab OR ‘patient flow’:ti,ab OR ‘patient turnover’:ti,ab OR caseload:ti,ab OR ‘case load’:ti,ab OR ‘case loads’:ti,ab OR caseloads:ti,ab OR throughput*:ti,ab OR ‘through put*’:ti,ab OR ‘patient journey’:ti,ab OR ‘patient inflow‘:ti,ab OR ‘patient path*’:ti,ab OR ‘patient disposition’:ti,ab OR ‘patient dispositions’:ti,ab OR bottleneck:ti,ab OR bottlenecks:ti,ab OR challenge:ti,ab OR challenges:ti,ab OR barriers:ti,ab OR barrier:ti,ab OR ‘Patient boarding’:ti,ab OR delay:ti,ab OR delays:ti,ab OR ‘choke point’:ti,ab OR ‘choke points’:ti,ab )

AND

(‘meta analysis’/de OR ‘systematic review’/de OR ‘systematic review’/exp OR ‘meta analysis’/exp OR ‘systematic review*’:ti,ab OR metaanal*:ti,ab OR ‘meta anal*’:ti,ab OR systematic:ti OR metasyn*:ti,ab OR ‘meta synth*’:ti,ab OR scoping:ti)

AND

(2018:py OR 2019:py OR 2020:py OR 2021:py OR 2022:py OR 2023:py) AND (‘article’/it OR ‘article in press’/it OR ‘review’/it)

('emergency service, hospital'/exp OR 'emergency service, hospital' OR 'emergency medicine'/exp OR 'emergency medicine' OR 'emergency medicine':ti OR 'emergency nursing'/exp OR 'emergency nursing' OR 'hospital emergency service'/exp OR 'hospital emergency service' OR 'hospital emergency services'/exp OR 'hospital emergency services' OR 'emergency hospital service'/exp OR 'emergency hospital service' OR 'emergency hospital services'/exp OR 'emergency hospital services' OR 'emergency department':ti OR 'emergency departments':ti OR 'emergency unit':ti OR 'emergency units':ti OR 'emergency ward':ti OR 'emergency wards':ti OR 'emergency room':ti OR 'emergency rooms':ti OR 'trauma center':ti OR 'trauma centers':ti OR 'trauma unit':ti OR 'trauma units':ti OR ('emergency treatment':ti AND hospital:ti)) AND ('crowding (area)'/exp OR 'crowding (area)' OR crowd*:ti,ab OR congest*:ti,ab OR overcrowd*:ti,ab OR gridlock*:ti,ab OR queu*:ti,ab OR overload*:ti,ab OR 'access block*':ti,ab OR 'patient flow':ti,ab OR 'patient turnover':ti,ab OR caseload:ti,ab OR 'case load':ti,ab OR 'case loads':ti,ab OR caseloads:ti,ab OR throughput*:ti,ab OR 'through put*':ti,ab OR 'patient journey':ti,ab OR 'patient inflow':ti,ab OR 'patient path*':ti,ab OR 'patient disposition':ti,ab OR 'patient dispositions':ti,ab OR bottleneck:ti,ab OR bottlenecks:ti,ab OR challenge:ti,ab OR challenges:ti,ab OR barriers:ti,ab OR barrier:ti,ab OR 'patient boarding':ti,ab OR delay:ti,ab OR delays:ti,ab OR 'choke point':ti,ab OR 'choke points':ti,ab) AND ('systematic review'/exp OR 'systematic review' OR 'meta analysis'/exp OR 'meta analysis' OR 'systematic review*':ti,ab OR metaanal*:ti,ab OR 'meta anal*':ti,ab OR systematic:ti OR metasyn*:ti,ab OR 'meta synth*':ti,ab OR scoping:ti)= 522

**CINAHL=225**

((MH "Emergency Service+") OR (MH "Emergency Medicine") OR (MH "Emergency Nursing+") OR (TI "emergency medicine" OR AB "emergency medicine") OR (TI "emergency nursing" OR AB "emergency nursing") OR (TI "Hospital Emergency Service" OR AB "Hospital Emergency Service") OR (TI "Hospital Emergency Services" OR AB "Hospital Emergency Services") OR (TI "Emergency Hospital Service" OR AB "Emergency Hospital Service") OR (TI "Emergency Hospital Services" OR AB "Emergency Hospital Services") OR (TI "Emergency Department" OR AB "Emergency Department") OR (TI "Emergency Departments" OR AB "Emergency Departments") OR (TI "Emergency Unit" OR AB "Emergency Unit") OR (TI "Emergency Units" OR AB "Emergency Units") OR (TI "Emergency Ward" OR AB "Emergency Ward") OR (TI "Emergency Wards" OR AB "Emergency Wards") OR (TI "Emergency Room" OR AB "Emergency Room") OR (TI "Emergency Rooms" OR AB "Emergency Rooms") OR (TI "trauma center" OR AB "trauma center") OR (TI "trauma centers" OR AB "trauma centers") OR (TI "trauma unit" OR AB "trauma unit") OR (TI "trauma units" OR AB "trauma units") OR ((TI emergency OR AB emergency) AND (TI hospital OR AB hospital)))

AND

((MH crowding+) OR (TI crowd* OR AB crowd*) OR (TI congest* OR AB congest*) OR (TI overcrowd* OR AB overcrowd*) OR (TI gridlock* OR AB gridlock*) OR (TI queu* OR AB queu*) OR (TI overload* OR AB overload*) OR (TI "access block*" OR AB "access block*") OR (TI "Patient flow" OR AB "Patient flow") OR (TI "Patient turnover" OR AB "Patient turnover") OR (TI "patient Caseload" OR AB "patient Caseload") OR (TI "Case Load" OR AB "Case Load") OR (TI "Case loads" OR AB "Case loads") OR (TI "patient Caseloads" OR AB "patient Caseloads") OR (TI "patient throughput*" OR AB "patient throughput*") OR "emergency department throughput" OR (TI through-put* OR AB through-put*) OR (TI "patient journey" OR AB "patient journey") OR (TI "patient path*" OR AB "patient path*") OR (TI "patient disposition" OR AB "patient disposition") OR (TI bottlenecks OR AB bottlenecks) OR (TI challenges OR AB challenges) OR (TI barriers OR AB barriers) OR (TI "Patient boarding" OR AB "Patient boarding"))

AND

(MH "Systematic Review") OR (MH "Meta Analysis") OR (TI "systematic review*" OR AB "systematic review*") OR (TI metaanal* OR AB metaanal*) OR (TI meta-anal* OR AB meta-anal*) OR (TI metasyn* OR AB metasyn*) OR (TI meta-synth* OR AB meta-synth*) OR (TI systematic) OR (TI scoping)) = 225

( ((MH "Emergency Service+") OR (MH "Emergency Medicine") OR (MH "Emergency Nursing+") OR (TI "emergency medicine" OR AB "emergency medicine") OR (TI "emergency nursing" OR AB "emergency nursing") OR (TI "Hospital Emergency Service" OR AB "Hospital Emergency Service") OR (TI "Hospital Emergency Services" OR AB "Hospital Emergency Services") OR (TI "Emergency Hospital Service" OR AB "Emergency Hospital Service") OR (TI "Emergency Hospital Services" OR AB "Emergency Hospital Services") OR (TI "Emergency Department" OR AB "Emergency Department") OR (TI "Emergency Departments" OR AB "Emergency Departments") OR (TI "Emergency Unit" OR AB "Emergency Unit") OR (TI "Emergency Units" OR AB "Emergency Units") OR (TI "Emergency Ward" OR AB "Emergency Ward") OR (TI "Emergency Wards" OR AB "Emergency Wards") OR (TI "Emergency Room" OR AB "Emergency Room") OR (TI "Emergency Rooms" OR AB "Emergency Rooms") OR (TI "trauma center" OR AB "trauma center") OR (TI "trauma centers" OR AB "trauma centers") OR (TI "trauma unit" OR AB "trauma unit") OR (TI "trauma units" OR AB "trauma units") OR ((TI emergency OR AB emergency) AND (TI hospital OR AB hospital))) ) AND ( ((MH crowding+) OR (TI crowd* OR AB crowd*) OR (TI congest* OR AB congest*) OR (TI overcrowd* OR AB overcrowd*) OR (TI gridlock* OR AB gridlock*) OR (TI queu* OR AB queu*) OR (TI overload* OR AB overload*) OR (TI "access block*" OR AB "access block*") OR (TI "Patient flow" OR AB "Patient flow") OR (TI "Patient turnover" OR AB "Patient turnover") OR (TI "patient Caseload" OR AB "patient Caseload") OR (TI "Case Load" OR AB "Case Load") OR (TI "Case loads" OR AB "Case loads") OR (TI "patient Caseloads" OR AB "patient Caseloads") OR (TI "patient throughput*" OR AB "patient throughput*") OR "emergency department throughput" OR (TI through-put* OR AB through-put*) OR (TI "patient journey" OR AB "patient journey") OR (TI "patient path*" OR AB "patient path*") OR (TI "patient disposition" OR AB "patient disposition") OR (TI bottlenecks OR AB bottlenecks) OR (TI challenges OR AB challenges) OR (TI barriers OR AB barriers) OR (TI "Patient boarding" OR AB "Patient boarding")) ) AND ( (MH "Systematic Review") OR (MH "Meta Analysis") OR (TI "systematic review*" OR AB "systematic review*") OR (TI metaanal* OR AB metaanal*) OR (TI meta-anal* OR AB meta-anal*) OR (TI metasyn* OR AB metasyn*) OR (TI meta-synth* OR AB meta-synth*) OR (TI systematic) OR (TI scoping)) ) --Limiters - Published Date: 20180101-

Expanders - Apply equivalent subjects- Search modes - Boolean/Phrase=225

**ISI WOS = 295**

(TS=("Emergency Service, Hospital") OR TS=("Emergency Medicine") OR ALL=("emergency nursing") OR ALL=("Hospital Emergency Service") OR ALL=("Hospital Emergency Services") OR ALL=("Emergency Hospital Service") OR ALL=("Emergency Hospital Services") OR TI=("Emergency Department") OR TI=("Emergency Departments") OR TI=("Emergency Unit") OR TI=("Emergency Units") OR TI=("Emergency Ward") OR TI=("Emergency Wards") OR TI=("Emergency Room") OR TI=("Emergency Rooms") OR TI=("trauma center") OR TI=("trauma centers") OR TI=("trauma unit") OR TI=("trauma units") OR (TI=(emergency) AND TI=(hospital)))

AND

(TS=(crowding) OR TI=(crowd*) OR AB=(crowd*) OR TI=(congest*) OR AB=(congest*) OR TI=(overcrowd*) OR AB=(overcrowd*) OR TI=(gridlock*) OR AB=(gridlock*) OR TI=(queu*) OR AB=(queu*) OR TI=(overload*) OR AB=(overload*) OR TI=("access block*") OR AB=("access block*") OR TI=("Patient flow") OR AB=("Patient flow") OR ALL=("patient inflow") OR TI=("Patient turnover") OR AB=("Patient turnover") OR TI=("patient Caseload") OR AB=("patient Caseload") OR TI=("patient Caseloads") OR AB=("patient Caseloads") OR TI=("patient throughput*") OR AB=("patient throughput*") OR TI=("emergency department throughput") OR AB=("emergency department throughput") OR TI=("patient journey") OR AB=("patient journey") OR ALL=("patient inflow") OR TI=("patient path*") OR AB=("patient path*") OR TI=("patient disposition") OR AB=("patient disposition") OR TI=("patient dispositions") OR AB=("patient dispositions") OR TI=(bottleneck) OR AB=(bottleneck) OR TI=(bottlenecks) OR AB=(bottlenecks) OR TI=(challenge) OR AB=(challenge) OR TI=(challenges) OR AB=(challenges) OR TI=(barriers) OR AB=(barriers) OR TI=(barrier) OR AB=(barrier) OR TI=("Patient boarding") OR AB=("Patient boarding") OR TI=(delay) OR AB=(delay) OR TI=(delays) OR AB=(delays) OR TI=("choke point") OR AB=("choke point") OR TI=("choke points") OR AB=("choke points"))

AND

(TS=("systematic reviews") OR TS=("meta analysis") OR TS=(Meta-Analysis) OR ALL=("Systematic Review") OR TI=("systematic review*") OR AB=("systematic review*") OR TI=(metaanal*) OR AB=(metaanal*) OR TI=(meta-anal*) OR AB=(meta-anal*) OR TI=(metasyn*) OR AB=(metasyn*) OR TI=(meta-synth*) OR AB=(meta-synth*) OR TS =(systematic) OR TI=(systematic) OR TI=(scoping))

(TS=("Emergency Service, Hospital") OR TS=("Emergency Medicine") OR ALL=("emergency nursing") OR ALL=("Hospital Emergency Service") OR ALL=("Hospital Emergency Services") OR ALL=("Emergency Hospital Service") OR ALL=("Emergency Hospital Services") OR TI=("Emergency Department") OR TI=("Emergency Departments") OR TI=("Emergency Unit") OR TI=("Emergency Units") OR TI=("Emergency Ward") OR TI=("Emergency Wards") OR TI=("Emergency Room") OR TI=("Emergency Rooms") OR TI=("trauma center") OR TI=("trauma centers") OR TI=("trauma unit") OR TI=("trauma units") OR (TI=(emergency) AND TI=(hospital))) AND (TS=(crowding) OR TI=(crowd*) OR AB=(crowd*) OR TI=(congest*) OR AB=(congest*) OR TI=(overcrowd*) OR AB=(overcrowd*) OR TI=(gridlock*) OR AB=(gridlock*) OR TI=(queu*) OR AB=(queu*) OR TI=(overload*) OR AB=(overload*) OR TI=("access block*") OR AB=("access block*") OR TI=("Patient flow") OR AB=("Patient flow") OR ALL=("patient inflow") OR TI=("Patient turnover") OR AB=("Patient turnover") OR TI=("patient Caseload") OR AB=("patient Caseload") OR TI=("patient Caseloads") OR AB=("patient Caseloads") OR TI=("patient throughput*") OR AB=("patient throughput*") OR TI=("emergency department throughput") OR AB=("emergency department throughput") OR TI=("patient journey") OR AB=("patient journey") OR ALL=("patient inflow") OR TI=("patient path*") OR AB=("patient path*") OR TI=("patient disposition") OR AB=("patient disposition") OR TI=("patient dispositions") OR AB=("patient dispositions") OR TI=(bottleneck) OR AB=(bottleneck) OR TI=(bottlenecks) OR AB=(bottlenecks) OR TI=(challenge) OR AB=(challenge) OR TI=(challenges) OR AB=(challenges) OR TI=(barriers) OR AB=(barriers) OR TI=(barrier) OR AB=(barrier) OR TI=("Patient boarding") OR AB=("Patient boarding") OR TI=(delay) OR AB=(delay) OR TI=(delays) OR AB=(delays) OR TI=("choke point") OR AB=("choke point") OR TI=("choke points") OR AB=("choke points")) AND (TS=("systematic reviews") OR TS=("meta analysis") OR TS=(Meta-Analysis) OR ALL=("Systematic Review") OR TI=("systematic review*") OR AB=("systematic review*") OR TI=(metaanal*) OR AB=(metaanal*) OR TI=(meta-anal*) OR AB=(meta-anal*) OR TI=(metasyn*) OR AB=(metasyn*) OR TI=(meta-synth*) OR AB=(meta-synth*) OR TS =(systematic) OR TI=(systematic) OR TI=(scoping)) – Publication Years: 2023 OR 2022 OR 2021 OR 2020 OR 2019 OR 2018 OR 2018 **= 295**

**Search in grey literature**

(government OR "health service website" OR report OR guideline OR “decision makers” OR policymakers) AND ("Emergency Service" OR "Emergency Medicine" OR "emergency nursing" OR "Hospital Emergency Service") AND "Patient flow"
