# Supplementary material for: Patient flow in emergency departments: a comprehensive umbrella review of solutions and challenges across the health system
Source: BMC Health Serv Res. 2024 Mar 5;24:274. doi: 10.1186/s12913-024-10725-6 (PMC10913567; doi:10.1186/s12913-024-10725-6)
Supplement: Supplementary file 3 — Supplementary Material 3. [file 12913_2024_10725_MOESM3_ESM.pdf]

Quality Assessment Template

Draft You saved about 2 hours ago.

Save as Draft

Publish

Item Settings

Editor

I want to start from scratch

1. Is the review question clearly and explicitly stated?
   1. –
      Yes
   2. –
      No
   3. –
      Unclear
   4. –
      Not applicable

2. Were the inclusion criteria appropriate for the review question?
   1. –
      Yes
   2. –
      No
   3. –
      Unclear
   4. –
      Not applicable

3. Was the search strategy appropriate?
   1. –
      Yes
   2. –

No

3. –

Unclear

4. –

Not applicable

4. Were the sources of studies adequate?

1. –

Yes

2. –

No

3. –

Unclear

4. –

Not applicable

5. Were the criteria for appraising studies appropriate?

1. –

Yes

2. –

No

3. –

Unclear

4. –

Not applicable

6. Was critical appraisal conducted by two or more reviewers independently?

1. –

Yes

2. –

No

3. –

Unclear

4. –

Not applicable

7. Were there methods to minimize errors in data extraction?

1. –
   Yes
2. –
   No
3. –
   Unclear
4. –
   Not applicable

8. Were the methods used to combine studies appropriate?

1. –
   Yes
2. –
   No
3. –
   Unclear
4. –
   Not applicable

9. Was the likelihood of publication bias assessed?

1. –
   Yes
2. –
   No
3. –
   Unclear
4. –
   Not applicable

10. Were recommendations for policy and/or practice supported by the reported data?

1. –
   Yes
2. –
   No
3. –
   Unclear

4. –

   Not applicable

11. Were the specific directives for new research appropriate?
    1. –

       Yes
    2. –

       No
    3. –

       Unclear
    4. –

       Not applicable

Preview

1. Is the review question clearly and explicitly stated?

Assess whether the review question is clearly and explicitly stated, and if it is formulated using the PICO elements to define the scope of the review and aid in the development of the search strategy. Determine if the question will aid both the review team in the conduct of the review and the reader in determining if the review has achieved its objectives. Note if the question is articulated in a published protocol or not.

1. Yes
2. No
3. Unclear
4. Not applicable

Extractors will also be able to add supporting text to justify their judgements
2. Were the inclusion criteria appropriate for the review question?

Evaluate whether the inclusion criteria for the review are appropriate and identifiable based on the review question. Ensure that the PICO elements are explicitly defined and that the criteria are detailed enough to make it clear which studies are eligible for inclusion. Take into account that some inclusion criteria may be specific to meta-analyses, such as the ability to conduct statistical analyses which would not be the norm for a systematic review. Check that the types of studies included are relevant to the review question, and exclude any studies that do not fit the inclusion criteria.

1. Yes
2. No
3. Unclear
4. Not applicable

Extractors will also be able to add supporting text to justify their judgements
3. Was the search strategy appropriate?

Assess whether the search strategy used in the systematic review was appropriate by examining the methods section or supplementary information. Check that the search strategy addressed each of the identifiable PICO components of the review question and included logical and relevant keywords, as well as subject headings and indexing terms. Consider any limits on the search, such as date or language, and determine if they were

appropriate and/or justified for the review question. Take into account the potential impact of any limits on the review findings, depending on the question being addressed.

1. Yes
2. No
3. Unclear
4. Not applicable

Extractors will also be able to add supporting text to justify their judgements
4. Were the sources of studies adequate?

Evaluate whether the sources of studies identified in the systematic review were adequate. Check that multiple electronic databases were searched, including major bibliographic citation databases such as MEDLINE and CINAHL. Ensure that other relevant databases were also searched, depending on the review question. Assess if trial registries were searched for reviews of effectiveness. Note if there was evidence of a comprehensive search for grey literature or unpublished studies, which may involve searching relevant websites or thesis repositories, to minimize publication bias.

1. Yes
2. No
3. Unclear
4. Not applicable

Extractors will also be able to add supporting text to justify their judgements
5. Were the criteria for appraising studies appropriate?

To answer this question, the reviewer should ensure that the systematic review presents a clear statement that critical appraisal was conducted, and the details of the items used to assess the included studies should be provided. The tools or instruments used for appraisal should be appropriate for the review question and the type of research conducted. For example, a systematic review of effectiveness should use a tool or instrument that addresses aspects of validity for experimental studies and randomized controlled trials, while a review assessing diagnostic test accuracy may use the QUADAS tool. This information may be presented in the methods section of the review, as an appendix, or as a reference to a source that can be located.

1. Yes
2. No
3. Unclear
4. Not applicable

Extractors will also be able to add supporting text to justify their judgements
6. Was critical appraisal conducted by two or more reviewers independently?

The systematic review should clearly state that critical appraisal was performed by at least two independent reviewers. This process should be detailed in the methods section of the review, including how disagreements between reviewers were resolved. The review should provide evidence of the inter-reviewer agreement, for example, by using Cohen's kappa or another appropriate measure. It is essential that critical appraisal is performed independently by two or more reviewers to minimize bias in the review process.

1. Yes
2. No
3. Unclear
4. Not applicable

Extractors will also be able to add supporting text to justify their judgements
7. Were there methods to minimize errors in data extraction?

To assess whether methods were used to minimize errors in data extraction, reviewers should look for evidence that the review team conducted data extraction in duplicate and independently. Reviewers should also check whether specific tools or instruments were used to guide data extraction and whether there was any evidence of piloting or training around their use. The systematic review should clearly describe the methods used for data extraction and how potential errors were addressed.

1. Yes
2. No
3. Unclear
4. Not applicable

Extractors will also be able to add supporting text to justify their judgements
8. Were the methods used to combine studies appropriate?

in a systematic review, the reviewer should assess if the synthesis of evidence presented is appropriate for the review question and type of review. If a meta-analysis has been conducted, the reviewer should evaluate if the studies were appropriately combined and if heterogeneity was assessed and explained. If a narrative synthesis was used, the reviewer should evaluate if it was appropriate for the included studies. In a qualitative review, the reviewer should assess if the methods used to synthesize findings are congruent with the review's methodology and if there is adequate descriptive and explanatory information to support the final findings.

1. Yes
2. No
3. Unclear
4. Not applicable

Extractors will also be able to add supporting text to justify their judgements
9. Was the likelihood of publication bias assessed?

To assess the likelihood of publication bias in a systematic review, the review should include a statement on whether this issue was considered and addressed. The review should describe any strategies used to minimize publication bias, such as a comprehensive search for all available evidence, including grey literature. The review should also assess the potential for publication bias statistically, such as by conducting Egger's test or using funnel plots. Any findings related to publication bias should be reported in the results and discussed in the interpretation of the review's findings.

1. Yes
2. No
3. Unclear
4. Not applicable

Extractors will also be able to add supporting text to justify their judgements
10. Were recommendations for policy and/or practice supported by the reported data?

To assess whether recommendations for policy and/or practice are supported by the reported data, the reviewer should look for a clear link between the results of the review and any recommendations made. The strength of the findings and the quality of the research should be considered in the formulation of recommendations. The recommendations should be based on the reported data and supported by the findings of the review.

1. Yes
2. No
3. Unclear
4. Not applicable

Extractors will also be able to add supporting text to justify their judgements
11. Were the specific directives for new research appropriate?

The systematic review process is recognised for its ability to identify where gaps in the research, or knowledge base, around a particular topic exist. Most systematic review authors will provide some indication, often in the discussion section of the report, of where future research direction should lie. Where evidence is scarce or sample sizes that support overall estimates of effect are small and effect estimates are imprecise, repeating similar research to those identified by the review may be called for and appropriate. In other instances, the case for new research questions to investigate the topic may be warranted.

1. Yes
2. No
3. Unclear
4. Not applicable

Extractors will also be able to add supporting text to justify their judgements

Feedback & Support

Find your answer, fast.

We've got a new Knowledge base that answers many frequently asked questions. Why not try that first?

View the knowledge base OR Send us an email
