# Supplementary material for: Patient flow in emergency departments: a comprehensive umbrella review of solutions and challenges across the health system
Source: BMC Health Serv Res. 2024 Mar 5;24:274. doi: 10.1186/s12913-024-10725-6 (PMC10913567; doi:10.1186/s12913-024-10725-6)
Supplement: Supplementary file 4 — Supplementary Material 4. [file 12913_2024_10725_MOESM4_ESM.pdf]

Preview

Study Details

Study ID

Title

Title of paper / abstract / report that data are extracted from

First Author

Year

Country in which the study conducted

1. ◯ United States
2. ◯ UK
3. ◯ Canada
4. ◯ Australia
5. ◯ Other

Aim of study

Objectives

Type of study/review

Participant description

The defining characteristics of the participants in studies included in the research syntheses

Total number of participants

The total number of participants from all studies included studies

Setting/context

Details of the setting of interest

Search Details

Sources searched

Where possible the names of databases and sources should be listed (i.e. if <5-10).

Range (years)

Range (years) of database searching and included studies. (If this is not readily identifiable in the table of study characteristics provided by the included synthesis, it should be discernable by scanning the date range of publications through the results section of the included review.

Number of studies included

Types of studies included

Country of origin of incl. studies

Appraisal

Appraisal instruments used

For the appraisal instruments or tools used to assess the risk of bias, cut-off score or any quality ranking should be reported.

Appraisal rating

For for checklist appraisals, reporting of cutt-off score or any ranking of quality should be reported

Analysis & Findings

Method of synthesis/analysis

The type of research synthesis as stated by the authors of the included review should be detailed.

Description of Interventions or strategies

Outcomes of the patient flow interventions

Challenges impeding patient flow

Root causes

Internal and external organization factors of inefficient patient flow

Outcomes of barriers/challenges

Barriers/challenges hampering the flow of patients

Other findings

These additional findings would provide a more detailed understanding of the factors that impact patient flow in EDs and help to identify effective interventions to improve patient outcomes.

Significance/direction

Heterogeneity

Comments

Important details regarding features of note about an included research synthesis, for example, are the conclusions of the included review consistent with the results presented by the study.
